# Isotopic variance among plant lipid homologues correlates with biodiversity patterns of their source communities

**DOI:** 10.1371/journal.pone.0212211

**Published:** 2019-02-27

**Authors:** Clayton R. Magill, Geoffrey Eglinton, Timothy I. Eglinton

**Affiliations:** 1 Lyell Centre, Heriot-Watt University, Edinburgh, United Kingdom; 2 Geological Institute, ETH Zürich, Zurich, Switzerland; 3 Department of Earth Sciences, University of Bristol, Bristol, United Kingdom; Wageningen University, NETHERLANDS

## Abstract

Plant diversity is important to human welfare worldwide, and this importance is exemplified in subtropical and tropical [(sub)tropical] African savannahs where regional biodiversity enhances the sustaining provision of basic ecosystem services available to millions of residents. Yet, there is a critical lack of knowledge about how savannahs respond to climate change. Here, we report the relationships between savannah vegetation structure, species richness, and bioclimatic variables as recorded by plant biochemical fossils, called biomarkers. Our analyses reveal that the stable carbon isotope composition (*δ*^13^C) of discrete sedimentary plant biomarkers reflects vegetation structure, but the isotopic range among plant biomarkers–which we call LEaf Wax Isotopic Spread (LEWIS)–reflects species richness. Analyses of individual biomarker *δ*^13^C values and LEWIS for downcore sediments recovered from southeast Africa reveal that the region’s species richness mirrored trends in atmospheric carbon dioxide concentration (*p*CO_2_) throughout the last 25,000 years. This suggests that increasing *p*CO_2_ levels during post-industrialization may prompt future declines in regional biodiversity (1–10 species per unit CO_2_ p.p.m.v.) through imminent habitat loss or extinction.

## Introduction

Observations and basic ecological theory provide corroborative support that diverse ecosystem services in savannahs are maintained by plant diversity [1, 2]. Yet, decades of research still leave some outstanding questions about African savannah dynamics during past intervals of dramatic climate changes (e.g., deglaciation) [3–5], and the consequences of human activities on regional biodiversity are a source of debate [6]. This debate continues, in part, because of lacking (paleo)biodiversity proxies applicable across multiple scales and key geologic archives [7]. For instance, although many (paleo)vegetation reconstructions employ pollen spectra as a metric of biodiversity [8], uncertainties surrounding pollen dispersal dynamics [9] and taphonomy [10] impose significant limitations on its use as a robust biodiversity proxy [7]. Additionally, pollen is largely reflective of fecundity as opposed to species abundance, distribution, and biomass [11]. As such, developing practical biodiversity proxies represents an urgent task for scientists and legislators alike as anthropogenic climate change intensifies [12].

Savannah vegetation communities are characterized by quick, nonlinear succession dynamics and threshold transitions [13] among grasses, woody plants and forbs [14–16]. The dominance of these coexisting plant functional types (PFTs) is reflected by physiognomic classifications of African savannah ecosystem structure as related to woody plant (crown) cover (c.f., Discussion A in S1 File), which defines understory gradients in resources [17], such as water, nutrients, and light [18, 19]. Ecosystem structure in turn influences underlying plant biodiversity patterns in savannahs [19, 20], but the nature of this influence is subject to differences in source accumulation area and time (i.e., scale) [21] and biogeographic variables, such as ecoregion extent [22, 23].

Conceptually, plant biodiversity is a measure of both species abundance (richness) and distribution (evenness) at the regional, landscape, and local level [21]. However, plant species richness (PSR) functions as a common index of biodiversity [1, 22] because of its strong positive relationships with resource-use differentiation [24] and functional trait divergence (e.g., growth habit) [14] among plants.

PFTs closely parallel functional trait divergence [25], which defines alternative strategies for mitigating pervasive resource constraints on and during plant development [26]. Associated functional traits include any phenotypic or chemical feature impacting plant fitness and their interactions with the environment [27], and thus describe vegetation dynamics with respect to ecophysiological factors [28] as opposed to strict taxonomic associations [14]. For instance, in southeast African savannah vegetation communities, a combination of divergent life strategies and taxonomic (i.e., species) richness account for most foliar chemical diversity [17], which in turn strongly parallels contemporary plant biodiversity [29]. As such, PFT-based techniques are reliably powerful for reconstructing plant biodiversity patterns alongside or in lieu of traditional taxonomic approaches (e.g., pollen spectra) [30].

Refractory plant biomarkers, such as most long-chain *n*-alkanes, in soils and terrestrial deposits exhibit molecular and isotopic signatures that reflect bioclimatic conditions during plant development [31]. Biomarker *n*-alkanes, in particular, are major molecular constituents of the waxy protective cuticle covering plant leaves [32, 33] that have characteristic odd-numbered C_27_–C_33_ homologue distributions in different PFTs [34]. High abundances of C_33_ *n*-alkanes (*n*-C_33_) characterize many grasses and forbs in modern savannahs [32], and higher *n*-C_27_ and *n*-C_29_ abundances characterize woody plants [33]. The stable carbon isotopic composition of biomarker *n*-alkanes, expressed here as *δ*^13^C values, are also characteristic in different PFTs, wherein C_4_ grasses have much higher values than woody plants and forbs with C_3_ photosynthesis [ca. *–*20‰ and –35±5‰, respectively (S1 Table)]. Among plants with C_3_ photosynthesis, biomarker *n*-alkane *δ*^13^C values decrease with higher resource availabilities and shade [35]. As such, biomarker *n*-alkane *δ*^13^C values serve as a uniquely quantitative reflection of PFT dominance [35], and thus ecosystem structure [16, 17], in savannahs across space and through time [36].

**Fig 1:**
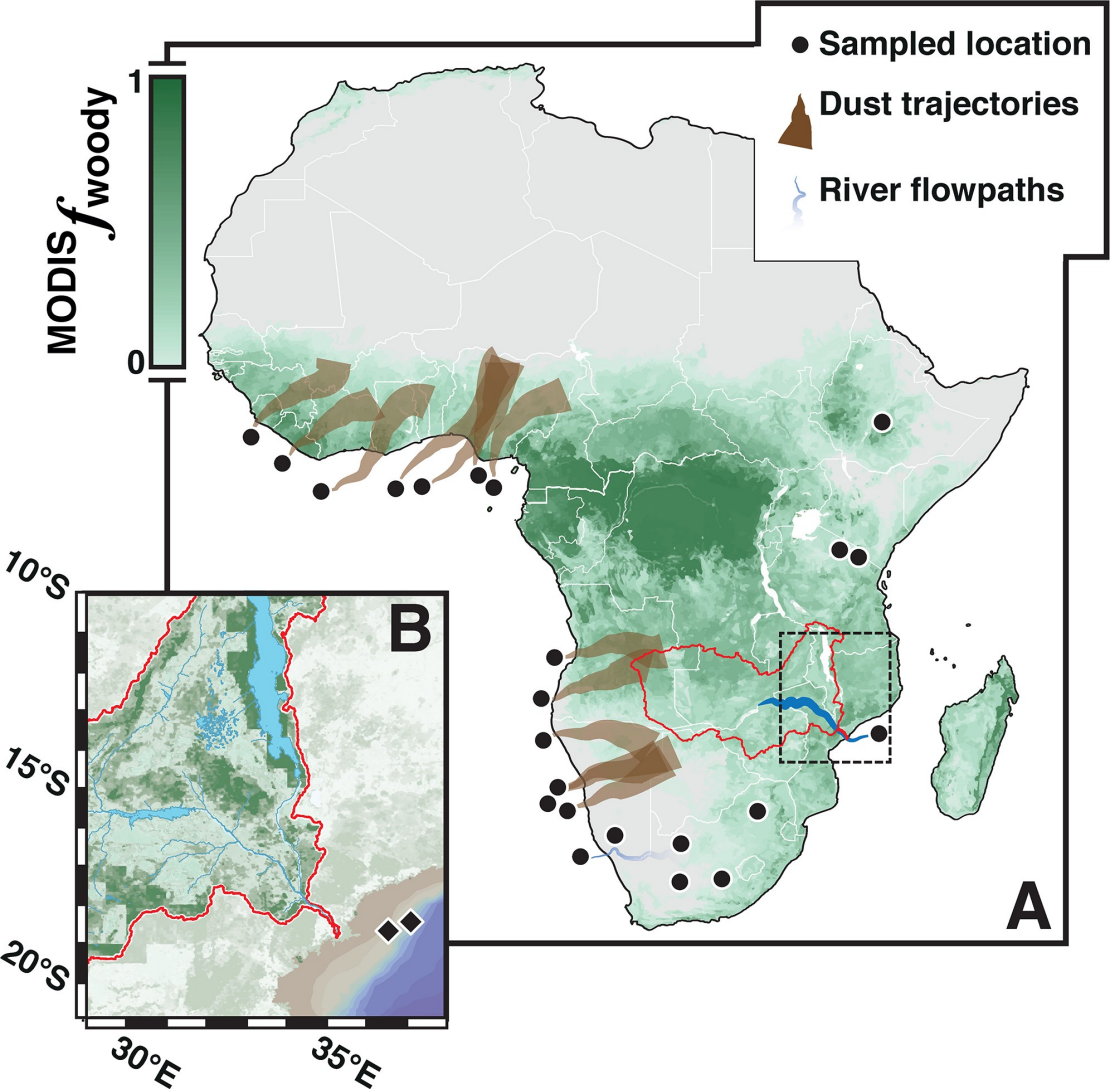
Satellite-estimated fractional tree cover and locations of the samples used for surface-sediment biomarker analyses (black circles). **A**, Satellite estimates of fractional tree cover (^MODIS^*f*_woody_) throughout (sub)tropical Africa [66]. Satellite data (available at landcover.org/data/) was resampled from 30-m resolution at 0.05° in ArcGIS 10.2.1 for improved figure visualization. Lighter-to-dark shading (green) represents increased fractional tree cover. Surface-sediment back-trajectories were constrained by geomorphological features, river discharge data, and Lagrangian atmospheric circulation models (Discussion D in S1 File). The Zambezi River catchment is outlined in red. **B**, Zoom-in of fractional tree cover estimates in the lower Zambezi sub-catchment (c.f., black dashed box in Fig 1A for map position). Black diamonds mark the marine sites used for sediment biomarker analyses: GeoB9307-3 [18°34.0`S, 37°22.9`E (542 m water depth)] [39], GIK16160 [18°14.5`S, 37°52.1`E (1339 m water depth)] [40].

We report here on a new approach for estimating (paleo)biodiversity in savannahs, developed from new and literature data on biomarker *n*-alkane signatures in contemporary plants, surface soils and terrestrial deposits derived from (sub)tropical African sources. This approach builds on earlier studies relating plant biodiversity with (i) tree cover [20], (ii) PFT dominance [15], and (iii) biomarker *n*-alkane signatures [35–38] to develop predictive relationships connecting plant species richness and biomarker *n*-alkane signatures in savannahs. Then, we extend these contemporary predictive relationships to marine sediment biomarker *n*-alkane records off the Zambezi River mouth (Fig 1) [39, 40] to reconstruct biodiversity patterns in southeast Africa since ~25 thousand years ago (kya).

## Materials and methods

### Contemporary plants

**Fig 2:**
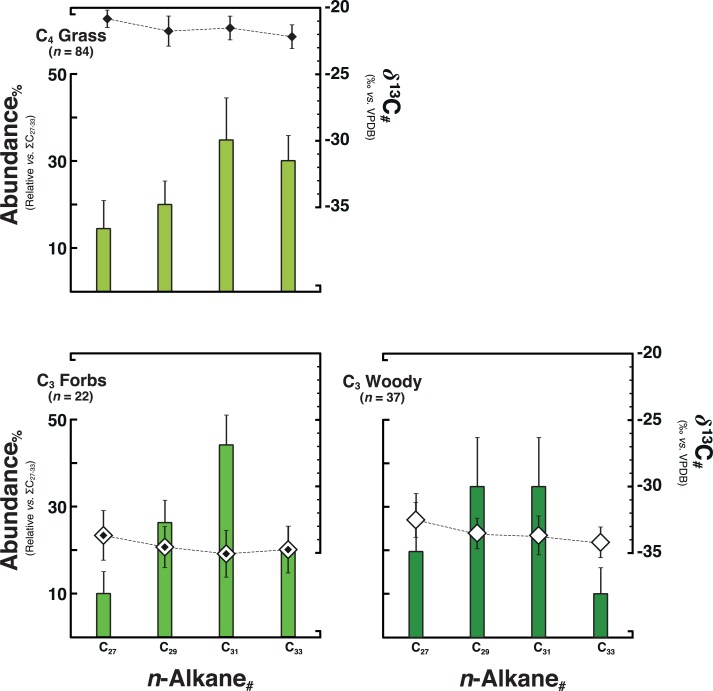
Compiled leaf-wax *n*-alkanes signatures of contemporary plants. Compiled leaf-wax *n*-alkanes signatures of 139 contemporary plants, which represent 82 distinctive species of (sub)tropical African savannah vegetation communities (S1 Table). Plants were separated by photosynthetic pathway and growth habit into one of three overarching plant functional types (PFTs): C_3_ woody (*n* = 42), C_3_ forbs (*n* = 22), and C_4_ grass (*n* = 75). Stable carbon isotopic values of C_27_–C_33_ *n*-alkanes (δ^13^C_#_) are shown as medians with their median absolute deviation (±MAD). Histograms depict the relative abundance of C_27_–C_33_ *n*-alkanes in each PFT (median±MAD).

We compiled both new and literature data of C_27_–C_33_ *n*-alkane *δ*^13^C values in contemporary plant leaves [*n* = 139 (Fig 2; S1 Table)] from at least 82 distinct species representative of the commonest African savannah vegetation communities (Discussion B in S1 File) [14–16]. To account for within and between-species isotopic variations caused by plasticity [41], appertaining plants were assigned to one of three dominant PFTs based on photosynthetic pathway and growth habit [15]: C_3_ woody plants (*n* = 42), C_3_ forbs (*n* = 22), and C_4_ grasses [*n* = 75 (Discussion B in S1 File)].

For new data, we collected fresh leaves and B-horizon soils during boreal summer of 2011 to complement the molecular and isotopic data reported by previous studies (S1 and S2 Tables). Fresh leaves [*n* = 44 (S1 Table)] were sampled from at Ngorongoro Conservation Area and the surrounding park (3.5±0.5°S, 35.0±1.0°E) together with correlative soil B-horizons [*n* = 11 (S2 Table)], which were sampled following protocols of Belsky *et al*. [17] from depths of 0–2.5 cm underneath surface soil (O/A horizon) layers. Corresponding permits were issued for regions around Olduvai Gorge (Arusha) by the Tanzania Wildlife Research Institute (TAWIRI) through the Commission for Science and Technology (COSTECH). Freeze-dried leaves or soils were mechanically powdered before accelerated solvent extraction (ASE) with dichloromethane:methanol [DCM:MeOH (85:15 v/v)] in a sequence of three cycles of 5 min at 10.3 MPa and 100°C with 70% flush volume [42]. Resultant total lipid extracts were evaporated to dryness under nitrogen, reconstituted in 50 μl of the extraction solvent, and then allowed to evaporate in a second ASE packed with 1 g quartz sand, 2 g of 5% (w/w) silver-impregnated silica gel, and 6 g activated silica gel [42]. Once evaporated, the unsaturated hydrocarbons (e.g., *n*-alkanes) were separated from associated total lipid extracts via selective, sequential ASE with hexane in one cycle of 1 min at 3.4 MPa and 50°C with 30% flush volume [42].

Once separated, the unsaturated hydrocarbons were characterized first by gas chromatography (GC) flame ionization detection on a HP 5890 [60-m HP5 (0.32 mm × 0.25 μm)]; GC temperature was set to 60°C for 1 min, ramped to 320°C at 6°C min^-1^, and held for an additional 20 min at 320°C. Thereafter, unsaturated hydrocarbons were measured by gas chromatography (combustion) isotope-ratio monitoring mass spectrometry on a Thermo TraceGC Ultra [60-m HP5 (0.32 mm × 0.25 μm)] and Thermo DeltaV Plus connected via a continuous flow interface using the same oven-ramp program as with GC-FID. Samples were injected in splitless mode and turned to carbon dioxide via combustion over nickel and platinum wire in helium at 1000°C. Stable carbon isotopic values are expressed in standard permil (‰) notation relative to Vienna Pee Dee Belemnite (VPDB):

$$\delta^{13}C=1000\left(\frac{R_{sample}}{R_{standard}}-1\right);\;R=\frac{{}^{13}C}{{}^{12}C}$$

Within-run precision (1σ) and accuracy were determined from co-injected lipids of known concentration and isotopic composition (c.f., Schimmelmann Standard B4) and have values of 0.11‰ and 0.10‰ (*n* = 88), respectively.

### Soils and terrestrial deposits

We also compiled biomarker *n*-alkane *δ*^13^C values (*n* = 40) for (sub)tropical African samples of terrestrial-derived fine-grain materials often incorporated into sedimentary geologic archives [i.e., sediments, soil, litter and dust; hereafter, “surface sediments” (S2 Table)]. These surface sediments were selected to reflect the major source-to-sink transport histories of biomarker *n*-alkanes (Discussions C and D in S1 File) [43] for (sub)tropical Africa and its spectrum of diverse savannah ecosystems, spanning grassland to woodland [16].

## Results

### Contemporary plants

Altogether, contemporary plants show significant differences in associated C_27_–C_33_ *n*-alkane *δ*^13^C (*δ*^13^C_27–33_) values within and between dominant PFTs (Fig 2). In contrast, the average range of *δ*^13^C_27–33_ values in individual leaves is about 1.9‰ for all the dominant PFTs (S1 Table). To represent this isotopic range across a homologous series of compounds, we introduce an index called LEaf-Wax Isotopic Spread (LEWIS):

$$LEWIS=max|\delta^{13}C_{x-y}|-min|\delta^{13}C_{x-y}|$$

Here, *δ*^13^C_*x*–*y*_ is representative of the *δ*^13^C values in a regular series of homologous compounds with *x* through *y* carbons (e.g., *δ*^13^C_27–33_ represents *n-*C_27_, *n-*C_29_, *n-*C_31_, and *n-*C_33_). In essence, associated LEWIS values represent the distribution-weighted PFT differences in carbon isotope discrimination among principle sources of biomarker *n*-alkanes in a sample [44], which is ideal for addressing plant functional trait divergence and biodiversity questions in complex ecosystems [45]. Although our study focuses on *n-*alkanes, in concept, LEWIS likewise could be applied to related homologous *n*-alkyl biomarkers, such as alcohols and fatty acids [37].

### Soils and terrestrial deposits

**Fig 3:**
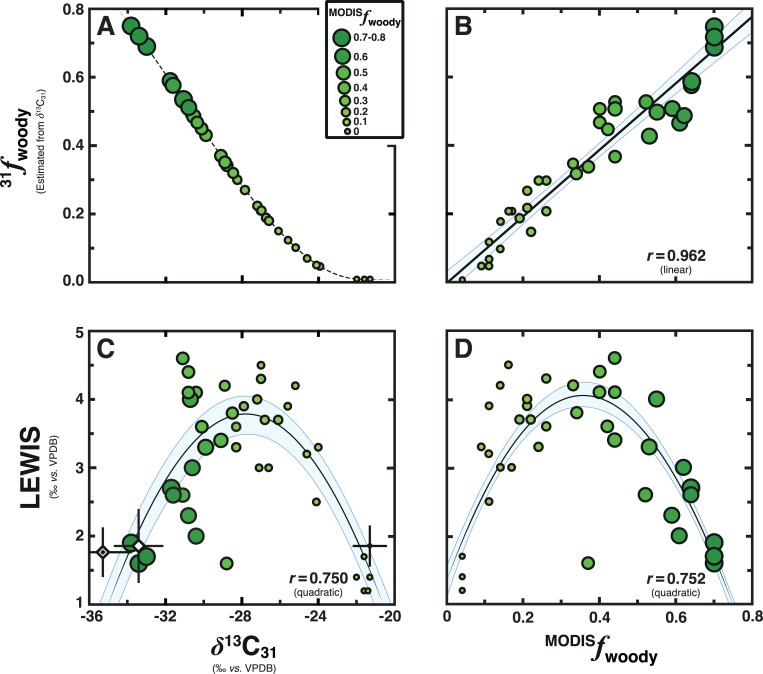
Bivariate relationships shared between surface-sediment LEWIS, *δ*^13^C_31_ values and fractional tree cover (^MODIS^*f*_woody_) estimates of (sub)tropical African source-region vegetation communities. Larger circle sizes and darker shading (green) both represent increased fractional tree cover (c.f., Fig 1). Light blue shaded bounds indicate empirical 90% confidence intervals as calculated from a Monte Carlo method [92]. Asymptotic significance (*p*-value) is less than 0.0001 for all the relationships shown. **A**, Biomarker reconstructions of fractional tree cover (^31^*f*_woody_) were calculated from *δ*^13^C_31_ values with a nonlinear equation [35]:
$${}^{31}f_{woody}=\{sin(-1.8353-0.08538\times \delta^{13}C_{31}){\}}^{2}$$
**B**, Biplot between satellite-estimated fractional tree cover (^MODIS^*f*_woody_) and biomarker-reconstructed fractional tree cover (^31^*f*_woody_). **C**, Relationship shared between surface-sediment LEWIS and *δ*^13^C_31_ values. Also shown are median values and the median absolute deviation of dominant PFTs (S1 Table) [15]: C_3_ woody plants [*n* = 37 (white diamond)], C_3_ forbs [*n* = 22 (double diamond)], and C_4_ grasses [*n* = 84 (black diamond)]. **D**, Relationship shared between surface-sediment LEWIS values and fractional tree cover (^MODIS^*f*_woody_) estimates.

Surface-sediment *δ*^13^C_27–33_ values show an inverse relationship with satellite and biomarker-estimated fractional tree cover of their source-vegetation communities (Fig 3). At first, *δ*^13^C_27–33_ values were compared to estimates of MODerate-resolution Imaging Spectradiometer (MODIS) derived fractional tree cover of their inceptive source-vegetation communities in (sub)tropical Africa [^MODIS^*f*_woody_ (Fig 1 and Discussion D in S1 File)]. Although estimates of fractional tree cover show a significant linear relationship with the *δ*^13^C values of C_27_–C_33_ biomarker *n*-alkanes as individual homologues (S2 Table), the relationship is strongest with *δ*^13^C values of *n*-C_31_ [*δ*^13^C_31_ (*r* = –0.939; *p* < 0.0001)]. Yet, the regression residuals of this relationship create a distinctive unimodal distribution, which tracks the characteristically quadratic rise of C_3_ forb biomass [19, 35] at intermediate levels of tree cover in savannahs [46]. To account for the effects of forbs on surface-sediment biomarker *n*-alkane signatures, we used *δ*^13^C_31_ values to derive more representative estimates (i.e., reconstructions) of fractional tree cover [^31^*f*_woody_ (Fig 3A and Discussion E in S1 File)]. Biomarker reconstructions of fractional tree cover show a much stronger correlation with satellite-estimated fractional tree cover as compared to individual *δ*^13^C_27–33_ values (Fig 3B):

$${}^{31}f_{woody}=0.940\;{}^{MODIS}f_{woody}+0.014(r^{2}=0.927)$$

Combined, these regression analyses indicate surface-sediment *δ*^13^C_31_ values reflect fractional tree cover in savannahs at timescales of biomarker *n*-alkane accumulation and turnover [47].

Surface-sediment LEWIS values show a wide range [~1.5–4.5‰ (Fig 3C and 3D)] that belies the consistent *δ*^13^C_27–33_ values of individual leaves (Fig 2). Associated LEWIS values show a distinctive unimodal relationship with *δ*^13^C_31_ values (Fig 3C) and ^MODIS^*f*_woody_ estimates (Fig 3D) that further indicates surface-sediment LEWIS does not simply parallel trends in reciprocal C_3_/C_4_ dominance or savannah fractional tree cover, respectively (Discussions E–G in S1 File). Considered together, such nonlinear relationships indicate surface-sediment LEWIS tracks an ecological threshold in savannah vegetation communities [13] related to ecosystem structure via canopy–gap gradients in carbon [35], water [48], and light [17, 19], which together can drive substantial differences in absolute *δ*^13^C_27–33_ values of individual leaves even within monospecific communities [49].

The unimodal relationship present between surface-sediment LEWIS values and fractional tree cover resembles closely patterns in resource variance (e.g., patch-scale spatial heterogeneities in carbon, water and light) along grassland-forest transitions [50]. In savannahs, resource variance increases with increased tree cover until it reaches a critical threshold [13]. Thereafter, resource variance decreases with further crown closure [19] alongside successive reductions in canopy–gap spatial heterogeneity [17, 50]. Although ecological thresholds are dynamic [16], resource variance in savannahs usually peaks at fractional tree cover of 0.25–0.45 [19, 24]. Given resource variance shows close correspondence with biodiversity patterns [16], it comes as no surprise that plant species richness in savannahs also usually peaks at fractional tree cover of ~0.35±0.10 (Figure A in S1 File) [19, 51].

**Fig 4:**
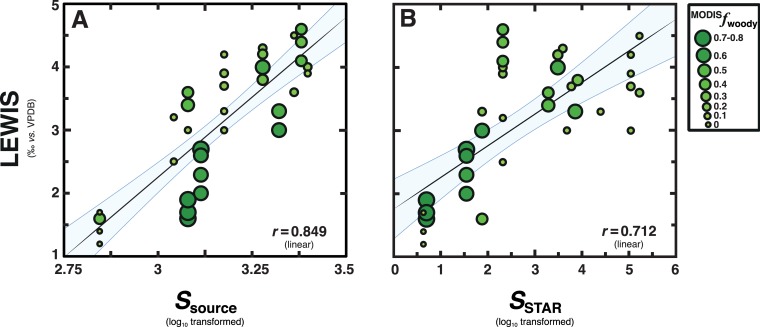
Bivariate relationships shared between surface-sediment LEWIS values and the corresponding plant species richness (PSR) of their source-region vegetation communities. Larger circle sizes and darker shading (green) both represent increased fractional tree cover (^MODIS^*f*_woody_) estimates (c.f., Fig 1). Solid lines represent linear regression models. Blue shaded bounds indicate empirical 90% confidence intervals as calculated from a Monte Carlo method [92]. Asymptotic significance (*p*-value) is less than 0.0001 for all the relationships shown. **A**, Biplot between surface-sediment LEWIS values and the ecoregion estimates of PSR (*S*_source_) [22] for their main source-region vegetation communities (Discussion F in S1 File). **B**, Biplot between surface-sediment LEWIS values and (re)scaled PSR estimates using predictive models of the species-time-area relationship (STAR) [23] for modern savannahs [*S*_STAR_ (Discussion F in S1 File)]. Note, we used log-transformed PSR for our analyses to improve data normality, minimize heteroscedasticity, and foster consistent linear relationships across disparate scales and datasets [1, 21, 23].

Surface-sediment LEWIS values show a significant linear correlation (*r* = 0.849; *p* < 0.0001) with literature estimates of PSR (*S*_source_) [22] for ecoregions of their main source-vegetation communities (Fig 4A and Discussion D in S1 File). Yet, the interpretive significance of this straight-line relationship is not immediately clear, as surface-sediments differ in “integration scale”, defined by generalizable average areas [i.e., accumulation extent (km^2^)] and times [i.e., sedimentation interval (kya)] for specific surface-sediment types [43, 52]. Therefore, we rescaled *S*_source_ estimates using predictive models of the species-time-area relationship (STAR) [23] for modern savannahs (Discussion F in S1 File), which functions as a standardization method for species richness in samples (i.e., sediment types) with incommensurate integration scales [53].

Rescaled *S*_source_ estimates–called *S*STAR−have a weaker correlation with surface-sediment LEWIS values as compared to simple ecoregion estimates of PSR (Fig 4B). Yet, the strength of this relationship grows much stronger after using partial bivariate regression models [54] to account for common covariance with fractional tree cover estimates [i.e., ^MODIS^*f*_woody_ (Figure C in S1 File)], which in turn co-varies with bioclimatic variables such as rain and fire regime [18]. The consistent linear correlations shared between surface-sediment LEWIS values and PSR, despite differences in respective integration scales [43] and transmission dynamics [52], support the premise that LEWIS functions as a semi-quantitative index of biodiversity patterns.

## Discussion

**Fig 5:**
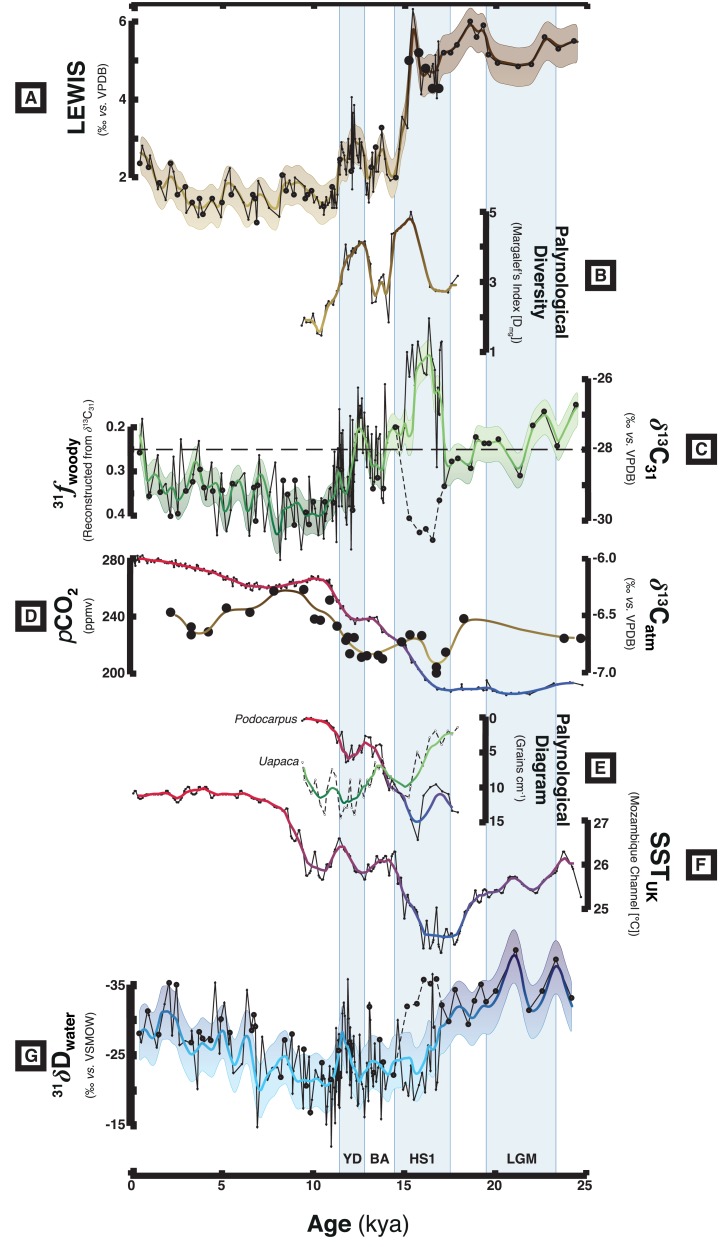
Downcore records of environmental change in southeast Africa over the last 25 kya. **A**, Sediment LEWIS (Discussion G in S1 File) for correlative marine cores recovered from off the Zambezi River mouth [GeoB9307 (filled circles) [39]; GIK16160 (open circles) [40]], which reflect the changing plant species richness of lower Zambezi vegetation communities [39]. Shaded bold lines show a combined 250-yr Gaussian smoothed time-series. Shaded bounds indicate empirical 90% confidence intervals as calculated from a Monte Carlo method [92]. **B**, Pollen-inferred biodiversity patterns at Lake Malawi [57] as shown with Margalef’s Index (D_mg_ = [*G*– 1][ln *N*]^–1^ where *G* is the cumulative number of taxa [e.g., genera] in a sample and *N* is the corresponding pollen sum) [63], which has a strong positive correlation with species richness at landscape scales [58, 62]. **C**, Downcore records of *δ*^13^C_31_ values, which are indicative of C_3_/C_4_ plant functional type dominance [35]. Associated *δ*^13^C_31_ values were also used to reconstruct fractional tree cover [^31^*f*_woody_ (dashed lines)] through time (Discussion D in S1 File). **D**, Atmospheric carbon dioxide concentrations (*p*CO_2_) and its stable carbon isotopic composition (*δ*^13^C_atm_) as recorded by polar ice [93], and the **E**, Pollen abundance diagram of *Podocarpus*, which is an indirect temperature indicator, and *Uapaca* and at Lake Malawi [57]. **F**, Downcore alkenone-estimates of the sea surface temperature (SST_UK_) for at GIK16160 [94], which is representative of temperature changes in the lower Zambezi sub-catchment [39]. **G**, Downcore records of the stable hydrogen isotopic composition in water used by plants [^31^*δ*D_water_ (Discussion E in S1 File)] reconstructed from *n*-C_31_*δ*D values in sediments at GeoB9307 (filled circles) [39] and GIK16160 (open circles) [40], representing precipitation changes in the lower Zambezi sub-catchment [39]. Lower ^31^*δ*D_water_ values are indicative of higher rainfall related to changes in monsoon intensity [48]. Abbreviations are written as: Younger Dryas (YD); Heinrich Stadial 1 (H1); Bolling-Allerød (BA); Last Glacial Maximum (LGM).

With a calibration surface-sediment LEWIS framework established, we sought to reconstruct Afrotropical biodiversity patterns during previous intervals of far-reaching global warming, such as the last deglaciation [~21–7 kya]. Therefore, we examined previously reported *δ*^13^C_27–33_ values in correlative marine cores recovered from off the mouth of the Zambezi River (Fig 1B) [39, 40], which is southern Africa’s second largest river system. Once combined, these records offer complementary perspectives on the developing plant biodiversity of the lower Zambezi sub-catchment (Discussions G–I in S1 File) throughout the last ~25 kya that transcends any particular record taken alone (Fig 5A and 5C).

Biomarker records from marine [39, 40] and lake sediments [55] suggest there was a gradual long-term expansion of trees and bushes in southeast Africa during the LGM–Holocene transition. In contrast, pollen records and beta diversity patterns suggest that the community phylogenetic structure of Zambezian ecoregions remained the same or similar since at least 25 kya [56–59]. In part, this seeming paradox may reflect the varied taxonomic resolution inherent to many pollen spectra [30]; though higher rank biodiversity parallels species richness under certain conditions, contemporary pollen spectra reflect (paleo)vegetation dynamics across a range of disparate area and time scales [60]. Consequently, pollen records can obscure important biodiversity patterns [53] and functional trait divergence at lower rank [61] that biomarker signatures can reveal by proxy [11, 55, 62]. This said, in our study, plant biodiversity is defined by published literature estimates of the observed taxonomic richness in specific (sub)tropical African ecoregions, which might demarcate expressly quantitative interpretations of LEWIS because ancient African savannahs might have differed in composition or structure as compared to modern analogues [5, 59]. Even still, the strong positive relationship species richness shows with sediment LEWIS forges a conceptual link between existing pollen and biomarker records, which allows us to differentiate changes in community production (e.g., plant biomass), ecosystem structure and biodiversity.

During glacial termination, about ~25–17.5 kya, reconstructed fractional tree cover (^31^*f*_woody_) estimates of 0.25±0.10 (Fig 5C) occur in conjunction with high pollen abundances among grasses and highland taxa (e.g., *Podocarpus* [Fig 5E]) [56]. High sediment LEWIS values also occur throughout this interval (Fig 5A) that, together with high corresponding palynological diversity (D_mg_) [63], denote species-rich vegetation communities with irregular crown cover and highly productive understory grasses, characteristic of African miombo woodlands [64].

High sediment LEWIS values and D_mg_ continued from ~17.5–15 kya (Fig 5A and 5B, respectively), but fractional tree cover estimates skewed to more extreme values (Fig 5C and Discussion H in S1 File). Amid Heinrich Stadial 1 [HS1 (17.5–14.7 kya)], the increasing pollen abundances of deciduous woodland taxa (e.g., *Uapaca* [Fig 5E]) [57, 58] indicate a shift toward Zambezian woodland-like vegetation communities [64], which are characterized by patches of dry-deciduous woody plants surrounded by grasses. Then, a dramatic decrease in sediment LEWIS values and D_mg_ occurs at the HS1–Bolling-Allerød (BA; 14.7–12.9 kya) transition alongside smaller increases in reconstructed fractional tree cover and the abundance of tropical forest taxa (e.g., *Macaranga*-type pollen) [58] together suggest the sudden expansion of less species-rich vegetation communities, such as mopane woodlands [22, 64].

Sediment LEWIS values and D_mg_ increase again around 12.9–11.7 kya [i.e., Younger Dryas (YD)], but low sediment LEWIS values during the earliest Holocene mirror reconstructed fractional tree cover and deciduous woodland taxa abundances [56, 57] that together are suggestive of the *Acacia*-*Combretum* woodlands [64] common in the lower Zambezi today [65]. The end-Holocene reconstructed fractional tree cover of 0.43 (S1 Table) is consistent with recent MODIS tree cover estimates of ~0.40 for the lower Zambezi sub-catchment (Discussion H in S1 File) [66, 67], and further supports our use of *δ*^13^C_31_ values as a reflection of PFT dominance and fractional tree cover.

The maximum isotopic difference demonstrated between end-member abundance-weighted *δ*^13^C_29_ and *δ*^13^C_33_ values of ~4.8‰ defines an internal benchmark of higher conceptual LEWIS index values apart from additional bioclimatic influences. Indeed, (micro)habitat heterogeneities in ecoregion structure could lead to additional differences of 3–4‰ between savannahs with homogenous PFT distributions as compared to relatively patchier ones [35]. This conceptual difference is corroborated by previous studies noting plant biodiversity parallels increasing patchiness even in savannahs with identical fractional tree cover estimates at the landscape scale (Discussion G in S1 File).

Beyond internal (micro)habitat influences on the carbon isotopic composition of leaf-waxes, major climatic controls on savannah vegetation communities are related to changes in rainfall, temperature, and *p*CO_2_ [68] though their relative importance can differ with observation scale [69]. For instance, equilibrium vegetation model (BIOME4) simulations [68] suggest that lower Zambezi ecosystem structure was controlled by *p*CO_2_ during glacial termination until ~10 kya (Fig 5D), but that temperature and, to a much lesser degree, rainfall together controlled Holocene succession (Fig 5F and 5G). These respective simulations are corroborative with the moderate correlation strength apparent between reconstructed fractional tree cover estimates (^31^*f*_woody_) and: (*i*) *p*CO_2_ during glacial termination and ~10 kya (*r* = 0.647), and (*ii*) reconstructed late-summer (austral) temperatures since ~10 kya (*r* = –0.714). Collectively, *p*CO_2_ and temperature explains 61% of the downcore variance in reconstructed fractional tree cover in a multiple regression model (Discussion I in S1 File).

Sediment LEWIS values share a relationship with corresponding *p*CO_2_ (*r* = –0.940) that does not become stronger in multiple regression models with a secondary predictor (Discussion I in S1 File). This correlation cannot be a simple consequence of increasing *p*CO_2_ on apparent C_3_ plant fractionation (Discussion G in S1 File) through the deglacial transition since such increases should have a negligible influence on carbon isotope discrimination in coincident C_4_ plants [70]. Rather, we suggest increasing *p*CO_2_ prompted decreasing plant functional trait divergence [71, 72] and, in turn, decreased biodiversity at the regional level [73]. For instance, increasing *p*CO_2_ promotes increased C_3_ photosynthetic water use efficiency [74], particularly plants with woody growth forms and forbs [75, 76], and thereby promotes decreased levels of facilitative-competitive interactions vis-à-vis resource-use strategies (i.e., water, carbon and light) [28, 77]. Resource-use strategies underlay plant functional trait divergence [77], which in turn underlies most differences in carbon isotope discrimination among populations (intraspecific) and between species [78, 79]. All things considered, this mechanism suggests increasing *p*CO_2_ prompts decreased LEWIS values as a consequence of decreased functional trait divergence (i.e., decreased PSR) and its effects on carbon isotope discrimination [79], which are related to resource use [41, 78]. Such a mechanism is consistent with the results of leaf-level function models [28], experimental data [80], and the consequences of increasing *p*CO_2_ on savannah biodiversity under modelled business-as-usual future climate scenarios within the next 100 years [81].

Even though these results indicate sediment LEWIS tracks changing plant biodiversity, there are some important limitations in our approach. For instance, isotopic responses of differing plant types are consequent to *interactive* effects of bioclimatic variables [68, 82], and furthermore are often species specific [83]. Indeed, plants show complex variation in their ecophysiological, biosynthetic, and molecular responsiveness to changing *p*CO_2_ over a wide range of timescales [76, 83]. These complexities underscore a caveat to our interpretations, as does recent literature about C_3_ plants re-assimilating photorespired CO_2_ [84], since each could have unpredictable effects on apparent landscape-scale carbon isotope discrimination. Our multivariate analyses also do not take into account the unpredictable effects of changing *p*CO_2_ on evolutionary processes [83] or environmental (historical) hysteresis [28], which can influence stable carbon isotopic composition of leaf biomass [82]. Even so, numerous studies suggest Quaternary plant biodiversity patterns were dominantly guided by (paleo)environmental influences on ecohydrological (functional) trait divergence [80, 85], suggesting post-LGM PSR trends are explicitly predictable using paired bioclimatic constraints and LEWIS.

In recent decades, intense debate has arisen about tropical biodiversity patterns during periods of far-reaching global warming, such as during glacial terminations and future climate scenarios [83]. This research develops a novel tool for reconstructions and, theoretically, projections of the species richness in savannah ecosystems at disparate area and time (i.e., integration) scales. Conservatively, *p*CO_2_ is modelled to reach 550–800 p.p.m.v. around 2050 and 2080, respectively [86]. The relative magnitude of this increase in concentration is comparable with the increasing *p*CO_2_ between about 17 kya and 10 kya (Fig 5D) that featured dramatic declines in species richness of Zambezi vegetation communities (Fig 5A and 5B). If the scaling (power) relationship present between species richness as compared to surface-sediment LEWIS (Fig 4) holds during past and future climate change events, anticipated impending *p*CO_2_ jumps will drive an estimated local loss of 1000±750 Zambezian species of flowering plants, which is at the extreme of terrestrial biodiversity privation estimates, on average, worldwide [87, 88]. Considering plant biodiversity exerts a direct influence on organic carbon storage [2] and terrestrial discharge [58] from drylands such as savannas [89], our results establish further motivation for in-depth investigation of the effects of future anthropogenic emission scenarios [81, 90]–particularly in relation to woody plant encroachment [91].

## Supporting information

S1 File This appendix contains supporting discussions (Discussions A–J) together with supporting figures (Figures A–F).
(PDF)

S1 Table Median and median absolute deviation values of C_27_–C_33_ *n*-alkane *δ*^13^C data and LEWIS values in contemporary plant leaves. (XLS)

S2 Table Surface-sediment locations alongside characteristics of their respective source (eco)regions, surface material terms, species–time–area relationship variables, measured C_27_–C_33_ *n*-alkane *δ*^13^C values, LEWIS values, and fractional tree cover estimates. (XLS)

S3 Table Measured biomarker *n*-alkane *δ*^13^C values and LEWIS values in sediment cores recovered from off the Zambezi River mouth. (XLS)
