# Supplementary material for: Isotopic variance among plant lipid homologues correlates with biodiversity patterns of their source communities
Source: PLoS One. 2019 Feb 27;14(2):e0212211. doi: 10.1371/journal.pone.0212211 (PMC6392421; doi:10.1371/journal.pone.0212211)
Supplement: S1 File — This appendix contains supporting discussions (Discussions A–J) together with supporting figures (Figures A–F). (PDF) [file pone.0212211.s001.pdf]

S1 File

Supporting discussions

Discussion A: Savannah classifications

African savannah ecosystems are defined by the irregular juxtaposition of woody plants on a continuous matrix of herbaceous groundcover [16]. Within the bounds of this broad definition, savannahs are subdivided on the basis of vegetation structure [35], particularly woody cover. Accordingly, we adopted the United Nations Educational, Scientific and Cultural Organization (UNESCO) classification system for savannah ecosystems (Fig B) [95]:

i. Forest: Over 70% woody cover (areal basis) with continuous crown canopy and occasional groundcover.
ii. Woodland: Between 40–70% woody cover with regular crown canopy and frequent groundcover.
iii. Wooded savannah: Between 10–40% woody cover with irregular crown canopy and common groundcover.
iv. Grassland: Less than 10% woody cover with abundant groundcover dominated by grasses.

In this study, we limit our consideration to savannah ecosystems with mean annual precipitation of less than 1200 mm [13, 96] because woody cover has a predictable response along precipitation gradients below this amount.

Discussion B: Contemporary plant leaves

We compiled both new and literature data [32, 33, 97-99] for leaf-wax *n*-alkanes of 139 contemporary plant leaves, which represent 82 distinctive species of (sub)tropical African savannah vegetation communities (S1 Table). Contemporary

plants were combined into dominant PFTs based on photosynthetic pathway and growth habit [15]: C_3 woody plants (*n* = 42), C_3 forbs (*n* = 22), and C_4 grasses (*n* = 75). Leaf-wax *n*-alkane signatures within individual PFTs (Fig 2 and S1 Table) are reported for median values ($\tilde{x}$) and the median absolute deviation (±MAD), which are robust measures of central tendency and dispersion, respectively [100].

Discussion C: Surface sediments

We compiled both new and literature data about C_{27}–C_{33} (biomarker) *n*-alkanes in (sub)tropical Africa surface soils and terrestrial sedimentary particles (hereafter referred to as "surface sediments") that include aeolian dust (*n* = 7), litter (*n* = 5) soil (*n* = 17), river and lake sediments (*n* = 3), and terrigenous component of ocean sediments [*n* = 8 (S2 Table)]. Literature data was compiled from earlier studies with δ^{13}C values reported for odd-numbered C_{27}–C_{33} *n*-alkanes derived from (sub)tropical African savannahs (c.f., Discussion A) because this range of homologues can establish uniquely parsimonious constraints on differential PFT contributions to surface sediments [32, 33] in spite of the inherent differences in wax production within and between dominant PFTs [34]. As an added benefit, this range can ameliorate most troublesome effects created by plants with variable carbon concentrating pathways (e.g., aquatic macrophytes) for surface-sediment LEWIS values as most (semi)aquatic vegetation synthesizes much lower concentrations of C_{27}–C_{33} *n*-alkanes as compared to terrestrial higher vegetation [101].

Discussion D: Satellite estimates of fractional woody cover

We used MODerate-resolution Imaging Spectroradiometer (MODIS) Vegetation Continuous Fields (VCF) tree cover composite datasets averaged from

2000 to 2010 [66] to develop gridded 30-m resolution [102] estimates of fractional tree cover ($^{MODIS}f_{woody}$) for (sub)tropical African savannahs (Fig 1). Previous research has demonstrated close correspondence between satellite-estimated fractional tree cover and local higher-resolution estimates of fractional tree cover [51, 103] despite marked differences in spatial- and thematic scale.

Gridded fractional tree cover estimates were integrated from across defining geographic source-region [43, 52] to calculate more representative ("rescaled") estimates of fractional tree cover. For soils specifically, gridded fractional tree cover estimates were averaged from across a radius of 60 m (~10,000 m^2) because soils incorporate organic matter within source-regions of 1000–10,000 m^2 [104] and the estimates were validated against high-resolution images (<5 m) from GoogleEarth to constrain sub-grid variation [105]. Geomorphological features were constrained for source-regions of 10^3–10^4 km^2 [43, 106] to calculate rescaled fractional tree cover estimates within individual fluviolacustrine catchments [107-110].

Precise source-regions of dust and terrigenous components of ocean sediments are difficult to constrain [110-113], but backwind trajectories [111] and river discharge data [109, 114] were used to calculate representative rescaled fractional tree cover estimates. We calculated 1500-km backwind trajectories during seasons with prevailing offshore winds [115] with Hybrid Single-Particle Lagrangian Integrated Trajectory (HYSPLIT) models [116]. Calculations are based on Global Data Assimilation System (GDAS) archive data for an altitude of 1500 m (~850 hPa) because most dust blown offshore of (sub)tropical Africa occurs in winds between 1000–2000 m altitude [113]. Indeed, dust from major source areas in western (sub)tropical Africa is entrained by shallow (500–1500 m) trade wind layers [111, 115]. Large scale monsoon convergence of West African near-surface air masses

uplift fresh leaf lipids abraded from vicinal terrestrial (last 100–1000 km of landside wind trajectories [115]) landscapes and transport them offshore [117, 118], such that dust-delivered lipids encode integrative abundance-weighted biomarker signals of their source-region's vegetation even if the mineral dust itself derives from a more remote area [115, 119, 120]. Aeolian organic matter also includes more minor contributions of lipids derived from soils and dessicated lake sediments, although these "old" lipids are usually quite similar as compared to aforementioned "fresh" lipids [115, 121] since average soil turnover rates in (sub)tropical Africa are short (~10–100 years) [47, 120].

Discussion E: Biomarker reconstructions of fractional woody cover

Previous studies reveal that biomarker *n*-alkane $\delta^{13}C$ values in surface sediments carry quantitative information about fractional tree cover when derived from savannahs [35, 122]. Because, on average, many plants in savannahs synthesize similar concentrations of *n*-C_{31} [34], we used the carbon isotopic composition of this *n*-alkane ($\delta^{13}C_{31}$) to reconstruct fractional tree cover [${}^{31}f_{\text{woody}}$ (Fig B)] [35]:

$$ {}^{31}f_{\text{woody}} = \{sin(-1.8353 - 0.08538 \times \delta^{13}C_{31})\}^2 $$

These reconstructions are representative of landscape-integrated fractional tree cover across a sedimentary plant biomarker source-region, as weighted by production (i.e., concentration) and the reciprocal transport distance (i.e., autocorrelation) [123]. Although reconstructed fractional tree cover (${}^{31}f_{\text{woody}}$) estimates and rescaled fractional tree cover (${}^{\text{MODIS}}f_{\text{woody}}$) estimates do not have any particular intrinsic relationship, our results demonstrate a strong positive correlation [m = 0.983, b = 0.01; *r* = 0.962 (Fig 4B)] between reconstructed and rescaled fractional tree cover.

Biomarker *n*-alkanes, in particular *n*-C_{31}, have δD values (e.g., δD_{31}) that are offset from, but highly correlated with, the δD value of their source-region rainfall (δD_{water}) [124]. Previous studies about African savannahs suggest that this offset between δD_{31} and δD_{water} values can be accounted for with $\delta^{13}C_{31}$ values [48]. In respective studies, $\delta^{13}C_{31}$ values were used to calculate offset (i.e., apparent fractionation) factors at the landscape scale [$\varepsilon_{landscape}$ (Fig E)] by pairing $^{31}f_{woody}$ estimates and the reciprocal fractional C_4 grass cover ($^{31}f_{grass} = 3.3 + 0.1 \times \delta^{13}C_{31}$) with literature values of the offset between δD_{31} values and the δD values of biosynthetic water sources (i.e., rainfall) for each of the corresponding plant functional types [C_4 grasses (–146‰); C_3 forbs (–124‰); C_3 woody plants (–109‰)] (Fig B). Then, $\varepsilon_{landscape}$ calculations were applied to ice-volume corrected δD_{31} values (Fig E) to reconstruct δD values of their source-region rainfall [$^{31}\delta D_{water}$ (Fig 5E)].

Discussion F: Estimating plant species richness

Here, we adopt the contemporary plant species richness (S_{source}) estimates of African ecoregions [22] as a representative index of plant biodiversity since associated S_{source} estimates minimize sample standardizations or conjecture [21]. Indeed, the relative ease with which savannah vegetation is differentiated by PFT and taxonomy justify published S_{source} estimates as a measure of plant biodiversity[119]. In our study, plant biodiversity is defined by published S_{source} estimates in selected (sub)tropical African ecoregions (S2 Table). Under such circumstances, plant biodiversity patterns will be relative as opposed to absolute, and thus restricted to specific ecosystems. Since modern savannahs also may not be analogous to savannahs of the past, we consider down-core sediment LEWIS trends as

representative of the relative changes as opposed to absolute differences in plant species richness.

Individual bioclimatic variables exert dynamic influences on species richness, which can differ with observation scale, across space and through time [23, 125]. However, numerous studies suggest that this scale dependence can be accounted for in savannahs with a single species-time-area relationship (STAR) [23, 126] because savannah vegetation communities are defined by plant–water–energy dynamics regardless of the scale used for observation [127]. For instance, contemporary plant species richness (PSR) estimates at the regional (100–10,000s km^2), landscape (1–100s km^2) and local level (0.01–1 km^2) have a strong positive relationship in savannahs [73] and throughout (sub)tropical Africa [128]. More importantly, previous studies suggest that S_{source} estimates, once rescaled, function as a reflection of local PSR [127, 128]. This is important for our work because it demonstrates the robustness of STAR for comparisons of PSR between disparate scales (e.g., 1 and 100 km^{-2} yr^{-1}) when rescaled to a standardized time-by-area unit (e.g., 10 km^{-2} yr^{-1}).

For our study, we rescaled S_{source} estimates [22] using predictive (slope) regression models [125, 126, 129] constrained by published STAR terms and African ecoregion characteristics (S2 Table):

$$S_{\mathrm{STAR}} = S_{\mathrm{source}} \left\{ \left(\frac{A_{\mathrm{source}}}{A_{\mathrm{STAR}}}\right)^{z} \left(\frac{1}{T_{\mathrm{STAR}}}\right)^{w} \left(\frac{A_{\mathrm{source}}}{A_{\mathrm{STAR}} T_{\mathrm{STAR}}}\right)^{u} \right\}^{-1}$$

S_{STAR} values represent the rescaled PSR (km^{-2} yr^{-1}). A_{source} values represent the contemporary geographic extent for a sample's inceptive ecoregion (km^2), and A_{STAR} values are representative of the source-region extent [67] for sedimentary plant biomarkers [km^2 (c.f., Discussion D and S2 Table)]. T_{STAR} values represent the average accumulation duration in years [43, 47] for each surface-sediment type. Exponential terms represent the slope of the species–area relationship (z), the species–

time relationship (w), and time-by-area interaction (u). The slope of the species–area relationship (SAR_z) was calculated from integration area [125]:

$$z = 0.264 + 0.0396\, log(A_{\mathrm{STAR}}) - 0.0021\, log(A_{\mathrm{STAR}})^2$$

The alternative use of a uniform z of 0.25 does not have a significant impact on regression slope and linear correlation strength when plotting partial linear residuals of S_{STAR} against LEWIS values, each on arcsin($\sqrt{{}^{\mathrm{MODIS}}f_{\mathrm{woody}}}$) (Discussion I). The slope of the species–time relationship (STR_w) was assumed to equal 0.40 in all instances [126, 129], as was the slope (u) of the interaction term [126]. Moreover, since all S_{STAR} values are derived from straightforward least-squares regression models, respective estimates should be semi-quantitative at least [130] and internally consistent [21] and therefore applicable to sedimentary records [131]. At length, S_{STAR} values were compared to current Biogeographical Information System on African Plant Diversity (BISAP) data (0.25×0.25-degree resolution) for gross validation [132] and baseline species richness assessments of Lower Zambezi ecosystems [133].

Discussion G: Potential drivers of high sediment LEWIS values

We use a simplified three end-member model that has equivalent total leaf tissue *n*-alkane concentrations in each dominant PFT (C_4 grasses, C_3 woody plants and C_3 forbs) [15] to understand how mixing proportions can influence sediment LEWIS independent from integration scale. As modelled, C_4 grasses represent a stable end-member since most African species show consistent biomarker *n*-alkane $\delta^{13}C$ values of –21.8±1.7‰ [n = 293 (S1 Table)] and higher relative abundances of *n*-C_{33} (30±10%). Woody plants and forbs show a wider range of biomarker *n*-alkane $\delta^{13}C$ values [–33.3±2.4‰ (n = 142) and –34.4±2.4‰ (n = 81), respectively] because of the influences of (micro)climate conditions (e.g., water availability and light level)

[134-136] on carbon isotopic fractionation among plants with C_3 photosynthesis [134], but both show much higher relative abundances of *n*-C_{29} (~30±10%). Importantly, previous studies suggest that biomarker *n*-alkanes usually peak at *n*-C_{31} (~40±10%) for dominant African savannah PFTs [34]. Further, our contemporary plant leaf data reveal that dominant African savannah PFTs also show near-identical LEWIS index values of ~1.9‰ (S1 Table). This consistent LEWIS index value in contemporary plant leaves creates a conceptual basis for interpretations of LEWIS in soils or sediments vis-à-vis mixing proportion models with finite unimodal distributions [5, 127].

Isotopic mass-balance calculations indicate *n*-C_{31} can be used to reconstruct PFT distribution abundance (i.e., cover) [35] because of its comparable concentrations in dominant African savannah PFTs and the characteristic relationship of $\delta^{13}C_{31}$ values against fractional tree cover (c.f., Fig 3) and C_4 grass cover. However, $\delta^{13}C_{29}$ and $\delta^{13}C_{33}$ values are skewed toward C_3 plants and C_4 grasses, respectively [122]. As a consequence, end-member abundance-weighted $\delta^{13}C_{29}$ and $\delta^{13}C_{33}$ values show differing quadratic (unimodal) trajectories against $\delta^{13}C_{31}$ values and demonstrate maximum isotopic difference at $\delta^{13}C_{31}$ values indicative of low variance (i.e., maximum evenness) in dominant PFT distribution abundances: f_{woody}, f_{grass} and f_{forb} estimates of 0.35, 0.40 and 0.25, respectively.

Precipitation exerts a consistently positive influence on apparent ^{13}C fractionation in contemporary plants [134]. This influence could lead to additional LEWIS differences of ~2‰ throughout the lower Zambezi, assuming precipitation averages range about 500–1500 mm within its catchment [137]. Altitude, in contrast, has a negative influence on contemporary plant ^{13}C fractionation [134] that translates into a maximal LEWIS difference of ~2‰ throughout the lower Zambezi (<2000

m.a.s.l. [65]). Considering precipitation correlates with altitude in southeast Africa [135, 138], their net influences on sediment LEWIS balance out.

Atmospheric carbon dioxide δ^{13}C values decreased <0.5‰ between about 25 kya and today (pre-industrial) [139], and furthermore do not develop parallel to core-sediment LEWIS. However, rising pCO_2 mirrors core-sediment LEWIS throughout this interval (Fig 5D). Although the specific mechanism(s) for such a relationship are debatable, we exclude any photosynthetic influences caused by pCO_2 because such changes would have a strictly positive influence on apparent ^{13}C fractionation of C_3 plants [140] and have a negligible negative influence on coeval C_4 plants [70]. Consequently, pCO_2 increases would lead to attenuated, low sediment LEWIS during periods with lower atmospheric carbon dioxide concentrations (e.g., glacial–deglacial transition) [141], and therefore cannot be a cause of the dramatic declines in core-sediment LEWIS between about 19.5–11.7 kya.

Discussion H: Down-core data integration

We integrated the *n*-alkane datasets of Schefuß et al. [39] and Wang et al. [40] for marine cores recovered from adjacent locations off the mouth of the Zambezi River (Fig 1):

GeoB9307-3 (18°34.0`S, 37°22.9`E; 542 m water depth)

GIK16160-3 (18°14.5`S, 37°52.1`E; 1339 m water depth)

Both cores were retrieved from zones with high (de)glacial–Holocene sedimentation of Zambezi-derived terrigenous material [114, 142]. The chronologies of both cores were previously established based on accelerator mass spectrometry (AMS) ^{14}C dates on mixed foraminifera, which were converted to calendar ages using CALIB 6.0 and

Marine09 calibration curve and a 405-yr reservoir age correction. Therefore, sample age-depth interpolations should be comparable between cores.

There are remarkably parallel trends in down-core sediment C_{27}–C_{33} *n*-alkane $\delta^{13}C$ and δD records (Figs D–E). However, earlier studies [40, 68] note a difference in respective molecular isotopic records amid Heinrich Stadial 1 (c.f., Fig 5), which was attributed [142, 143] to a stronger influence of hinterland tributaries at GIK16160 than at GeoB9307 (Fig F). This difference is absent in our core-sediment LEWIS record, which we consider as meaning plant biomarkers were discharged from rivers in a single ecoregion or in multiple ecoregions with similar source-vegetation communities (e.g., eastern, southern, and Zambezian miombo woodlands) [144]. This consideration is consistent with the similar species in ecoregions of the so-called *Zambezian domain* [144, 145], despite notable differences in estimated tree cover and leaf biomass [103, 146]. Source-vegetation communities derived from a single or similar ecoregions is also consistent with river-dominated transmission of Zambezian-derived terrigenous matter (e.g., plant biomarkers and detrital lithogenic clays [142, 147] to coastal Mozambique margin sites [39, 148] because near all the rivers that discharge into coastal Mozambique margin waters flow through Zambezian miombo woodlands [133] within their final 100s km overland flow [64] such that local, intermittent differences in sub-basin sources [148] would have a nominal influence on interpretations of down-core sediment LEWIS due to transport mixing [142] despite more uncertain effects on individual biomarker *n*-alkane $\delta^{13}C$ records.

Previous studies suggest that Zambezi river suspension contributed ~40-80% of the downcore clay particles at Mozambique margin sites during glacial termination, about 20–10 kya [142, 143]. Although changes in inferred Zambezi clay particle contributions show some similarities with core-sediment LEWIS trends amid this

interval (Fig F), there is an inverse relationship such that high Zambezi clay particle contributions occur when core-sediment LEWIS has much lower values. This relationship is counterintuitive, since one would theoretically presume core-sediment LEWIS featured higher values when derived from multiple clay provenances. We suggest that differences in clay provenance would have a nominal influence on core-sediment LEWIS because of the widespread Zambezi floristic affinity present throughout the southeast African region [95], which features a shared Quaternary phylogenetic and biogeographical history [59]. This suggestion is supported by parallels in core-sediment LEWIS trends and the coinciding palynological diversity given at Lake Malawi (Fig 5E), which would be unaffected by perturbations in clay provenance at GeoB9307-3 and GIK16160-3 [114, 142]. Although both records could be influenced by geographic changes in catchment basin accumulation area [114, 148], rescaling (power) relationships in sedimentary processes [43, 52] and during proxy propagation in marine and fluviolacustrine systems [149] make simultaneous changes improbable.

Explicit terrestrial (air) temperature estimates during glacial termination and the mid-Holocene are scant for southeast Africa [150, 151], but there is a strong parallel between mean annual lower Zambezi air temperature and local Indian Ocean sea-surface temperature (SST) [39, 152]. Therefore, we used Mozambique Channel SSTs reconstructed from alkenones (SST_{UK}) [94] to reconstruct Lower Zambezi temperature changes (Fig 5D). Although these reconstructed temperature changes are relative, our multivariate regression models are robust to differences in absolute value and their explanatory power will be unchanged [100].

Discussion I: Regression analyses

We assessed bivariate relationships between estimated fractional tree cover ($^{MODIS}f_{woody}$) and biomarker *n*-alkane signatures using ordinary (partial) least-squares regression with the statistical *R* (http://CRAN.R-project.org) computing packages named {*lmer4*}, {*mgcv*} and {*vegan*}. Regressions use arcsin($\sqrt{^{MODIS}f_{woody}}$) – a statistical transformation used to improve linearity in ecological datasets [100] – as the explanatory (predictor) variable. Bivariate regression models were used to assess secondary predictor influences on surface-sediment LEWIS (S3 Table) by plotting partial (linear) residuals of LEWIS from its regression on arcsin($\sqrt{^{MODIS}f_{woody}}$) against those of S_{source} and S_{STAR} residuals on arcsin[$\sqrt{^{MODIS}f_{woody}}$ (Fig C)]. The strong parallel, linear relationships of the corresponding partial residual plots emphasize the significance of the relationship shared between LEWIS and PSR in spite of differences in sedimentary properties and time–area integration scale. Even so, "sediment type" (S2 Table) has significant higher-order explanatory power (R_1^2 = 0.203–0.246) as a random variable (slopes and intercept) during predictive multilevel hierarchical linear regression analyses [53] of S_{source} and S_{STAR} and their residuals on arcsin($\sqrt{^{MODIS}f_{woody}}$) that highlight the significance of deposition dynamics (e.g., proxy propagation rates and turnover [149]) independent from simple species-time-area relationships [126] and fractional tree cover. It is important to understand that this significance does not confound the interpretation of down-core records unless a record features incommensurate sediment types.

We resampled down-core records [LEWIS, $\delta^{13}C_{31}$ ($^{31}f_{woody}$), pCO_2, SST_{UK}, and $^{31}\delta D_{water}$] at binned 250-yr steps to create uniform interpolated time-series, and then compared them with Fourier cross-correlation. Considering pCO_2 had the highest linear correlation coefficient (r = –0.940) with LEWIS during single-factor analyses, all further multiple regression models were constrained to include pCO_2. This

common constraint befits observations of the influence pCO_2 has on modern savannah vegetation communities [153] and the inferred importance of pCO_2 on (sub)tropical African succession since at least 25 kya [68]. Since carbon dioxide concentrations can correlate with other important bioclimatic variables, we assessed the independent influence of these secondary predictors on core-sediment LEWIS by partial (multivariate) regression models, which account for covariance among predictor variables. Only partial regression models with SST_{UK} and $^{31}\delta D_{water}$ as secondary predictors showed higher explanatory power as compared to only pCO_2 for variation in core-sediment LEWIS. The combination of pCO_2, SST_{UK}, and $^{31}\delta D_{water}$ together account for about 90% of the variance shown in core-sediment LEWIS between 25 kya and the Common Era ($r = 0.947$).

Discussion J: Zambezi River catchment

The Zambezi River originates in western Zambia (1450 m.a.s.l.) and flows almost 3000 km southeast before it discharges into the western Indian Ocean (Mozambique Channel). In conjunction with an extensive network of tributaries, the Zambezi River catchment drains a cumulative area of 1,570,000 km^2 [154], which is separated by geomorphic characteristics into three sub-catchments: the Upper, Middle, and Lower [155]. Here, our discussion will focus on the distal Lower Zambezi sub-catchment (Fig 1B) because it comprises the endmost ~500 km of the main watercourse [64], and exerts a major influence on discharge material to near-shore sediments of the coastal Mozambique Channel [142, 143].

The majority of Lower Zambezi discharge is derived from one of two main rivers – the Shire and Luangwa – and hydrodynamic models suggest there is nominal transfer of Upper and Middle sub-basin sediments into downriver reaches of the

Lower Zambezi [156, 157]. Although earlier studies suggest that Cyperaceae could have caused increased $\delta^{13}C_{31}$ values in discharged Zambezian organic matter amid deglaciation (Fig 5C) [39], C_4 sedges (e.g., papyrus) likely give a nominal influence on our interpretations of LEWIS because dissolved organic carbon derived from macrophytes is usually quite low in rivers as compared to coeval terrestrial taxa, and – based on modern observations and historical data – decreases during periods of drier conditions [158]. Thus, although there will be some uncertainties in absolute reconstructed biodiversity patterns, our approach should be internally consistent and likewise captures systematic changes in ecoregion estimates of both raw and (re)scaled taxonomic richness estimates among phytozones of (sub)tropical Africa (Fig 4) [22].

Supporting tables

S1 Table: Median and median absolute deviation (MAD) values of C_{27}–C_{33} *n*-alkane δ^{13}C data and LEWIS values in contemporary plant leaves [32, 33, 97-99] in three overarching plant functional types (PFTs) for savannahs [15]: C_3 woody plants (n = 42), C_3 forbs (n = 22), and C_4 grasses (n = 75). Important taxonomic distinctions within each PFT are also shown. In accordance with convention, average chain length (ACL) and weighted-mean average δ^{13}C ($\delta^{13}C_{WMA}$) were characterized:

$$\mathrm{ACL} = \sum\left\{\frac{x\,\mathrm{C}_x}{\mathrm{C}_x}\right\}$$

$$\delta^{13}\mathrm{C}_{\mathrm{WMA}} = \sum\left\{\frac{\delta^{13}\mathrm{C}_x\,\mathrm{C}_x}{\mathrm{C}_x}\right\}$$

Nomenclature refers to the concentration (C_x) or composition ($\delta^{13}C_x$) of an *n*-alkane homologue with x carbons.

S2 Table: Surface-sediment locations alongside characteristics of their respective source (eco)regions, surface material terms, species–time–area relationship variables, measured C_{27}–C_{33} *n*-alkane δ^{13}C values, LEWIS values, and fractional tree cover (${}^{\mathrm{MODIS}}f_{\mathrm{woody}}$ and ${}^{31}f_{\mathrm{woody}}$) estimates.

The composition, age and distribution of terrestrial biomarkers in soils or sediments are entwined with the source(s) and transformation of these compounds during progressive source-to-sink transmission [43, 106, 121]. This in turn is influenced by particle mobilization and transport dynamics [149]. Although sedimentary particles are often defined by physicochemical features (e.g., grain size or roundness), we use more mechanistic definitions vis-à-vis integration area and transport histories to define seven sediment types (Discussion F): dust, litter, soil, lake, river and marine sediments with abundant terrigenous material from riverine or wind-blown sources.

We use ecoregion names as defined by global WWF terminology (http://www.worldwildlife.org/science/data/terreco.cfm). Soil and terrestrial-derived sediment ("surface sediments") types were assigned tentative surface integration areas (A_{STAR}) and timescales of formation [T_{STAR} (Discussion F)]. We rescaled S_{source} estimates using predictive models of the species-time-area relationship (Discussion F) for modern savannahs. The slope (z) of the species–area relationship (SAR_z) was calculated from integration area [125]:

$$z = 0.264 + 0.0396\ log(A_{\mathrm{STAR}}) - 0.0021\ log(A_{\mathrm{STAR}})^2$$

The slope (*w*) of the species–time relationship (STR_w) was assumed to equal 0.40 in all instances [129], as was the slope (*u*) of the interaction term [126], which is essential for accurate rescaled biodiversity predictions [69].

S3 Table: Measured biomarker *n*-alkane $\delta^{13}C$ values and LEWIS values derived from data reported by Schefuß et al. [39] and Wang et al. [40] for sediment cores recovered from off the Zambezi River mouth (Discussion H).

Supporting figures

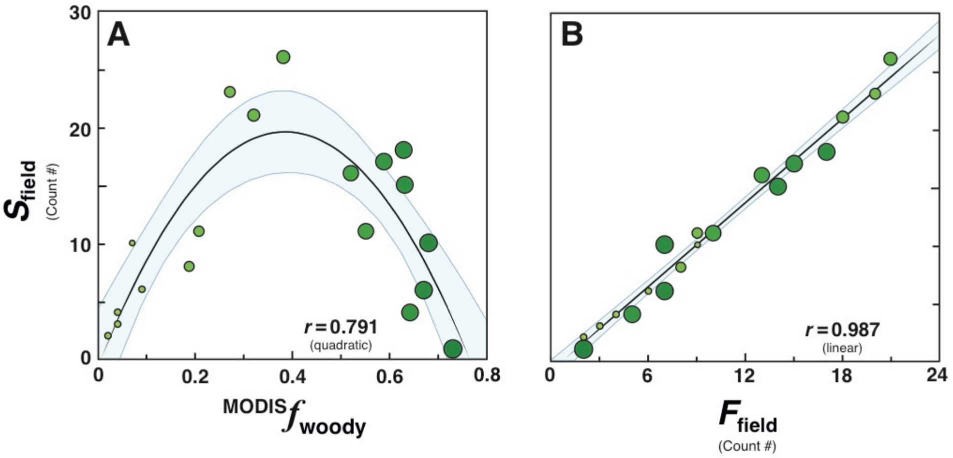

Fig. A: Visual (field) counts of plant species richness (S_{field}) against (**A**) moderate-resolution imaging spectroradiometer estimates of fractional woody cover (${}^{\text{MODIS}}f_{\text{woody}}$) at 30-m resolution [102] over 250 m^2 transect plots in the lower Zambezi sub-catchment [144], and (**B**) counts of the corresponding plant functional richness (F_{field}) [159] for identical transect plots [144]. Associated F_{field} counts are derived from empirical distinctions apparent between relevant functional traits in co-occuring plants (e.g., growth form and habit) [159]. Larger circle sizes and darker shading (green) both represent increased fractional tree cover (c.f., Fig 1). Blue shaded bounds indicate empirical 90% confidence intervals as calculated from a Monte Carlo method [92]. Asymptotic significance (p-value) is less than 0.0001 for all the relationships shown.

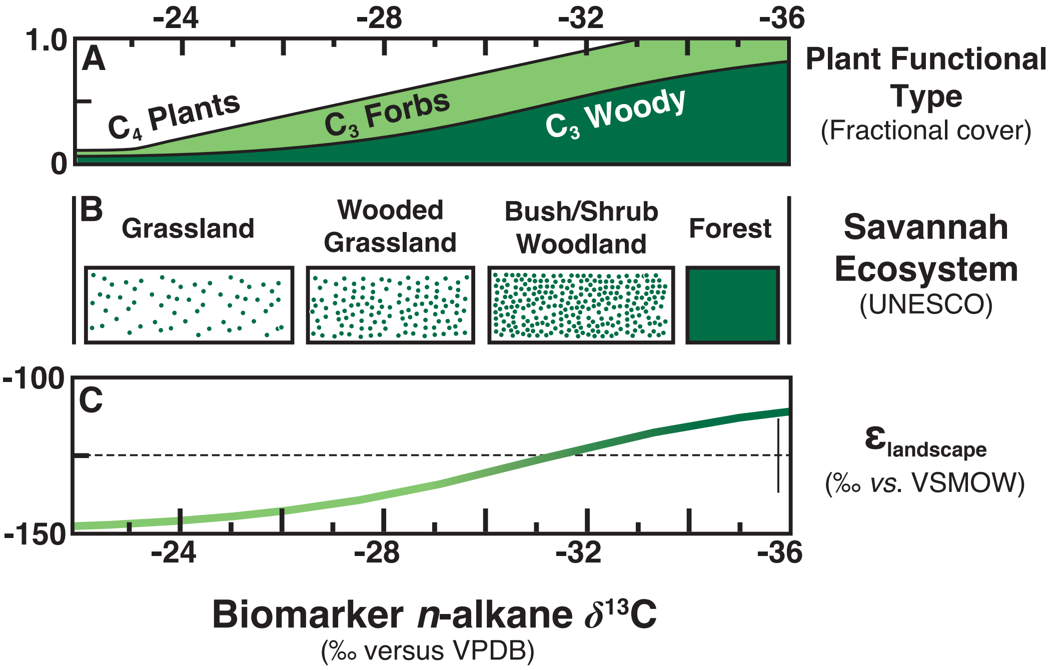

Fig. B: Schematic depiction of how individual biomarker *n*-alkane δ^{13}C values are related to ecosystem structure and landscape deuterium offset factors ($\varepsilon_{landscape}$) as functions of fractional tree cover (c.f., Discussion E). **A**, Sediment $\delta^{13}C_{31}$ values were used to reconstruct the cover of three overarching plant functional types[14] (PFTs): C_4 grasses, C_3 forbs, and C_3 woody plants. Fractional tree cover ($^{31}f_{woody}$) was reconstructed from the equation [35]:

$$^{31}f_{woody} = \{sin(-1.8353 - 0.08538 \times \delta^{13}C_{31})\}^2$$

The fraction of C_3 forbs was assumed to equal the difference between $^{31}f_{woody}$ and reconstructed C_4 grass cover ($3.3 + 0.1 \times \delta^{13}C_{31}$ [35]). **B**, Fractional tree cover reconstructions were related to savannah ecosystem structure with United Nations Educational, Scientific, and Cultural (UNESCO) terminology [95]. **C**, At length, biomarker *n*-alkane δ^{13}C values (i.e., $\delta^{13}C_{31}$) were used to calculate $\varepsilon_{landscape}$ values by pairing plant functional type reconstructions with literature values of the characteristic offset between δD_{31} values and the δD values of biosynthetic water sources in each PFT (i.e., isotopic mass-balance) [48].

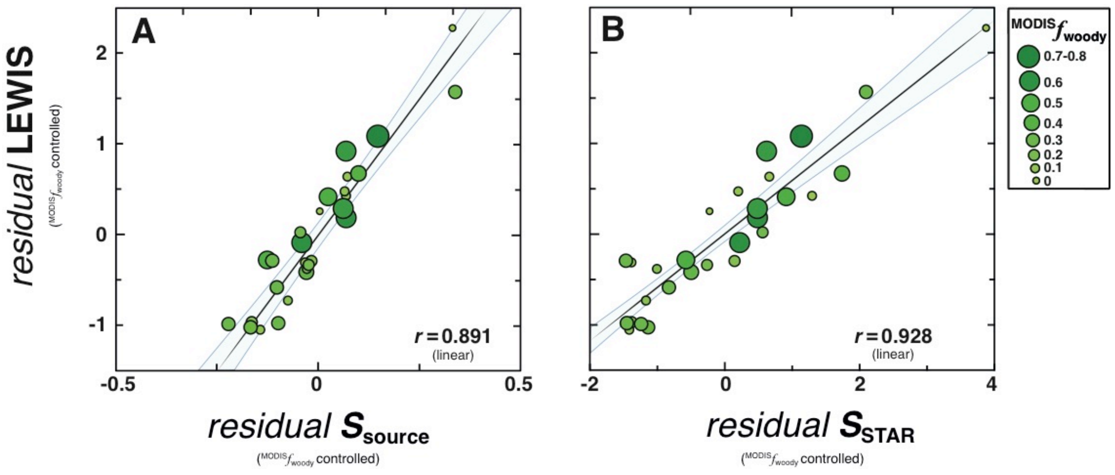

Fig. C: Partial (linear) residual plots of surface-sediment LEWIS against (**A**) the contemporary plant species richness estimates of African ecoregions (S_{source}) and (**B**) time–area rescaled S_{source} estimates [S_{STAR} (S2 Table)] as modeled by predictive species–time–area relationships (Discussion F). Residuals were calculated from respective single-factor regression models on arcsin($\sqrt{^{\text{MODIS}}f_{\text{woody}}}$) to account for their common covariance with fractional tree cover. Larger circle sizes and darker shading (green) both represent increased fractional tree cover (c.f., Fig 1). Blue shaded bounds indicate empirical 90% confidence intervals as calculated from a Monte Carlo method [92]. Asymptotic significance (p-value) is less than 0.0001 for all the relationships shown.

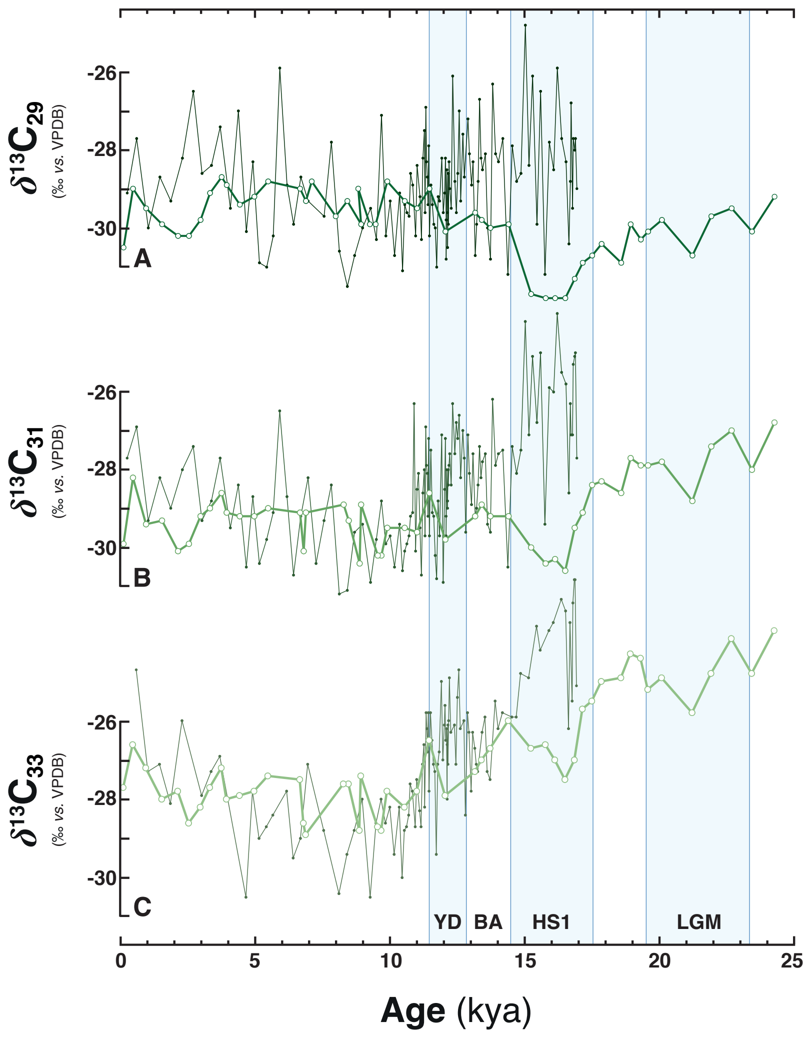

Fig. D: Core-sediment δ^{13}C values of biomarker *n*-alkanes: **A**, *n*-C_{29} ($\delta^{13}C_{29}$); **B**, *n*-C_{31} ($\delta^{13}C_{31}$); and **C**, *n*-C_{33} ($\delta^{13}C_{33}$) for data compiled from Schefuß et al. [39] (filled circles) and Wang et al. [40] (open circles). Because *n*-C_{27} data were sometimes unavailable, we corrected for $\delta^{13}C_{27}$ values with a 2nd degree polynomial function ($y = 0.056\,x^2 + 0.530\,x + 0.749$) based on relationships apparent for down-core sediment $\delta^{13}C_{29}$ and $\delta^{13}C_{31}$ values of in Wang et al. [40]. Core-sediment LEWIS values (i.e., C_{27}–C_{33} *n*-alkanes) and the corrected C_{29}–C_{33} *n*-alkane values show a very strong linear relationship (m = 0.954; b = 0.076; $r = 0.996$; *p*-value < 0.0001). Abbreviations are written as: Younger Dryas (YD); Heinrich Stadial 1 (H1); Bolling-Allerød (BA); Last Glacial Maximum (LGM).

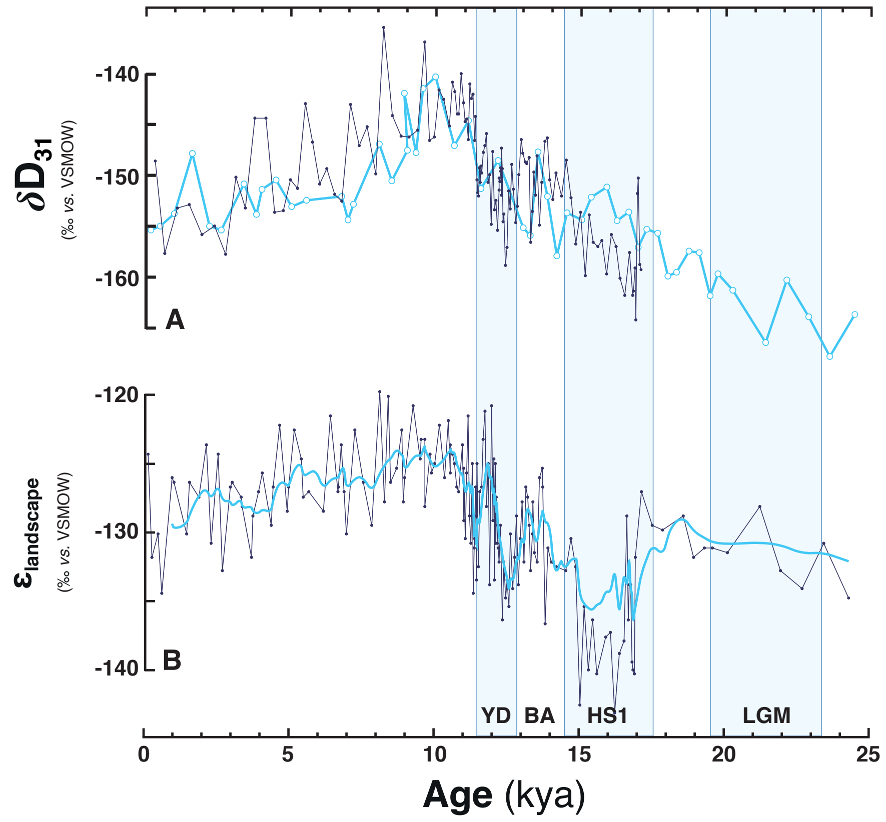

Fig. E: Downcore records of hydroclimate change in southeast Africa over the last 25 kya. **A**, Down-core sediment δD values of *n*-C_{31} (δD_{31}) from Schefuß et al. [39] (filled circles) and Wang et al. [40] (open circles). **B**, Core-sediment $\delta^{13}C_{31}$ values were used to calculate apparent landscape fractionation factors ($\varepsilon_{landscape}$) by paired fractional tree cover reconstructions ($^{31}f_{woody}$) and the reciprocal fraction of C_3 forb and C_4 grass cover [35] with literature values of the offset between measured $\delta^{13}C_{31}$ values and the δD values of biosynthetic water sources in dominant PFTs (Discussion E). Bold lines show a continuous averaged 5-pt timeseries. Abbreviations are written as: Younger Dryas (YD); Heinrich Stadial 1 (H1); Bolling-Allerød (BA); Last Glacial Maximum (LGM).

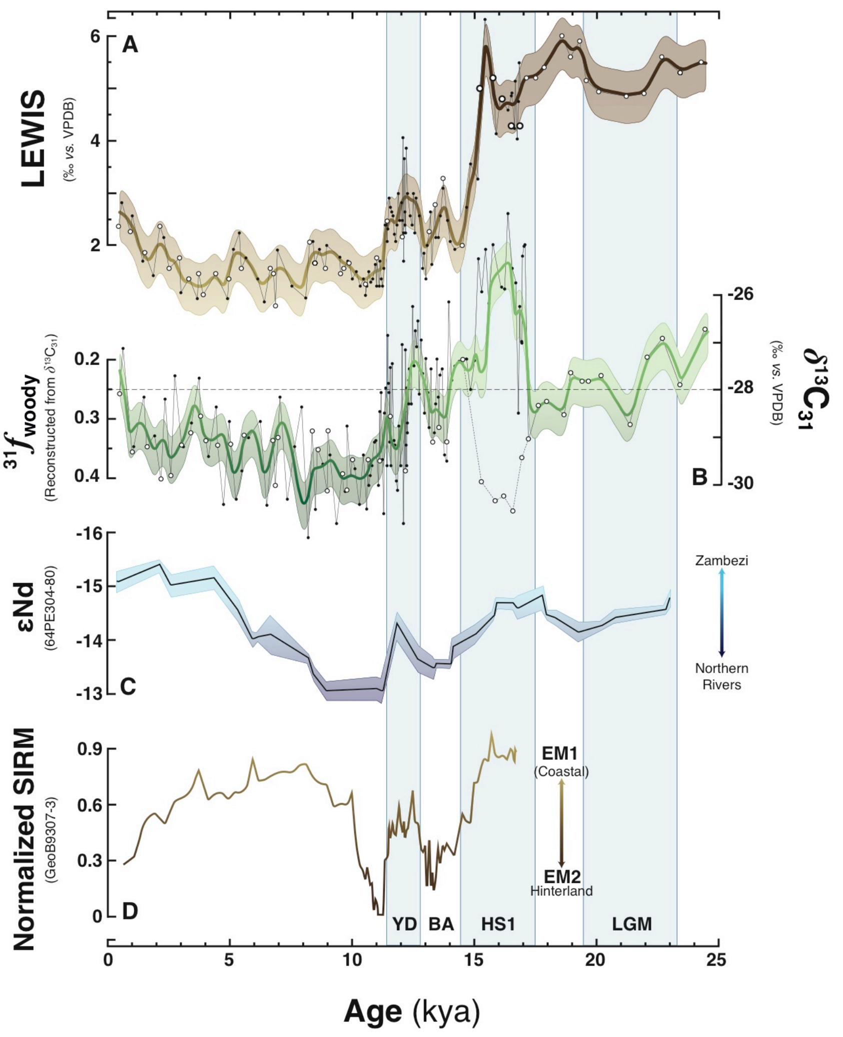

Fig. F: Downcore records of environmental change in southeast Africa over the last 25 kya. **A**, Core-sediment LEWIS (Discussion H) for correlative marine cores recovered from off the Zambezi River mouth [GeoB9307 (filled circles) [39]; GIK16160 (open circles) [40]], which reflect the changing plant species richness of lower Zambezi vegetation communities [39]. Shaded bold lines show a combined 250-yr Gaussian-smoothed time-series. Shaded bounds indicate empirical 90% confidence intervals as calculated from a Monte Carlo method [92]. **B**, Downcore records of $\delta^{13}C_{31}$ values from at GeoB9307 (filled circles) [39] and GIK16160 (open circles) [40], which are indicative of C_3/C_4 plant functional type dominance [39]. Associated $\delta^{13}C_{31}$ values were also used to reconstruct fractional tree cover [$^{31}f_{woody}$ (dashed lines)] [35] through time. **C**, Neodymium isotopic signatures (εNd) of the clay fraction of sediments recovered from near GIK16160-3 [142] that document changes in sediment provenance. Higher values indicate increased contribution of clays from the Zambezi River, and lower values indicate more northern river (e.g., Licungo) clay contributions. **D**,

Normalized (relative) contributions of total remnant magnetization (SIRM) from end-member acquisition curves on magnetic mineral concentrations [148]. End-member 1 (EM1) derives from coastal regions of the Zambezi catchment, but EM2 derives from the hinterland.

Supporting references (N.B., both main [1-94] and supporting [95-159] references are included herein for comprehensiveness)

1. Hooper DU, Solan M, Symstad A, Diaz S, Gessner MO, Buchmann N, et al. Species diversity, functional diversity and ecosystem functioning. In: Inchausti P, Loreau M, Naeem S, editors. Biodiversity and Ecosystem Functioning: Synthesis and Perspectives Oxford: Oxford University Press Oxford, UK; 2002. p. 195-208.
2. Maestre FT, Quero JL, Gotelli NJ, Escudero A, Ochoa V, Delgado-Baquerizo M, et al. Plant species richness and ecosystem multifunctionality in global drylands. Science. 2012; 335(6065):214-8.
3. Valentini R, Arneth A, Bombelli A, Castaldi S, Cazzolla Gatti R, Chevallier F, et al. A full greenhouse gases budget of Africa: synthesis, uncertainties, and vulnerabilities. Biogeosciences. 2014;11(2):381-407. doi: 10.5194/bg-11-381-2014.
4. Calvo MM, Prentice IC. Effects of fire and CO_2 on biogeography and primary production in glacial and modern climates. New Phytol. 2015;208(3):987-94.
5. Maguire KC, Nieto-Lugilde D, Fitzpatrick MC, Williams JW, Blois JL. Modeling species and community responses to past, present, and future episodes of climatic and ecological change. Annu Rev Ecol Evol S. 2015;46:343-68.
6. Broennimann O, Thuiller W, Hughes G, Midgley GF, Alkemade JMR, Guisan A. Do geographic distribution, niche property and life form explain plants' vulnerability to global change? Glob Change Biol. 2006;12(6):1079-93.
7. Loreau M, Naeem S, P. Inchausti J. P. Grime, Hooper D. Raffaelli, Inchausti P, Bengtsson J. Biodiversity and Ecosystem Functioning: Current Knowledge and Future Challenges. Science. 2001;294(5543):804-8. doi: 10.1126/science.1064088.
8. Birks HJB, Felde VA, Bjune AE, Grytnes J-A, Seppä H, Giesecke T. Does pollen-assemblage richness reflect floristic richness? A review of recent developments and future challenges. Rev Palaeobot Palyno. 2016;228:1-25.
9. Goring S, Lacourse T, Pellatt MG, Mathewes RW. Pollen assemblage richness does not reflect regional plant species richness: a cautionary tale. J Ecol. 2013;101(5):1137-45.
10. Campbell ID. Quaternary pollen taphonomy: examples of differential redeposition and differential preservation. Palaeogeogr Palaeocl. 1999;149(1):245-56.
11. Castañeda IS, Caley T, Dupont L, Kim J-H, Malaizé B, Schouten S. Middle to Late Pleistocene vegetation and climate change in subtropical southern East Africa. Earth Planet Sc Lett. 2016;450:306-16.
12. Koellner T, de Baan L, Beck T, Brandão M, Civit B, Margni M, et al. UNEP-SETAC guideline on global land use impact assessment on biodiversity and ecosystem services in LCA. The International Journal of Life Cycle Assessment. 2013;18(6):1188-202. doi: 10.1007/s11367-013-0579-z.
13. Hoffmann WA, Geiger EL, Gotsch SG, Rossatto DR, Silva LCR, Lau OL, et al. Ecological thresholds at the savanna-forest boundary: how plant traits, resources and fire govern the distribution of tropical biomes. Ecol Lett. 2012;15(7):759-68. doi: 10.1111/j.1461-0248.2012.01789.x.
14. Cowling RM, Esler KJ, Midgley GF, Honig MA. Plant functional diversity, species diversity and climate in arid and semi-arid southern Africa. J Arid Environ. 1994;27(2):141-58. doi: 10.1006/jare.1994.1054.
15. Skarpe C. Plant functional types and climate in a southern African savanna. J Veg Sci. 1996;7(3):397-404.
16. House JI, Archer S, Breshears DD, Scholes RJ. Conundrums in mixed woody–herbaceous plant systems. J Biogeogr. 2003;30(11):1763-77.

17. Belsky AJ, Amundson RG, Duxbury JM. The effects of trees on their physical, chemical and biological environments in a semi-arid savanna in Kenya. J Appl Ecol. 1989;26:1005-24.
18. Peterson DW, Reich PB. Fire frequency and tree canopy structure influence plant species diversity in a forest-grassland ecotone. Plant Ecol. 2007;194(1):5-16. doi: 10.1007/s11258-007-9270-4.
19. Soliveres S, Maestre FT, Eldridge DJ, Delgado-Baquerizo M, Quero JL, Bowker MA, et al. Plant diversity and ecosystem multifunctionality peak at intermediate levels of woody cover in global drylands. Global Ecol Biogeogr. 2014;23(12):1408-16. doi: 10.1111/geb.12215.
20. Shirima DD, Totland Ø, Munishi PKT, Moe SR. Relationships between tree species richness, evenness and aboveground carbon storage in montane forests and miombo woodlands of Tanzania. Basic Appl Ecol. 2015;16(3):239-49.
21. Whittaker RJ, Willis KJ, Field R. Scale and species richness: towards a general theory of species diversity hierarchical. J Biogeogr. 2001;28(4):453-70. doi: 10.1046/j.1365-2699.2001.00563.x.
22. Kier G, Mutke J, Dinerstein E, Ricketts TH, Küper W, Kreft H, et al. Global patterns of plant diversity and floristic knowledge. J Biogeogr. 2005;32(7):1107-16. doi: 10.1111/j.1365-2699.2005.01272.x.
23. Scheiner SM, Chiarucci A, Fox GA, Helmus MR, McGlinn DJ, Willig MR. The underpinnings of the relationship of species richness with space and time. Ecol Monogr. 2011;81(2):195-213.
24. Araya YN, Silvertown J, Gowing DJ, McConway KJ, Peter Linder H, Midgley G. A fundamental, eco-hydrological basis for niche segregation in plant communities. New Phytol. 2011;189(1):253-8. doi: 10.1111/j.1469-8137.2010.03475.x.
25. Sala OE, Lauenroth WK, Golluscio RA. Plant functional types in temperate semi-arid regions. In: Smith TM, Shugart HH, Woodward FI, editors. Plant functional types: their relevance to ecosystem properties and global change: Cambridge University Press; 1997. p. 217-33.
26. Roscher C, Schumacher J, Lipowsky A, Gubsch M, Weigelt A, Pompe S, et al. A functional trait-based approach to understand community assembly and diversity–productivity relationships over 7 years in experimental grasslands. Perspect Plant Ecol. 2013;15(3):139-49.
27. Mitchell N, Moore TE, Mollmann HK, Carlson JE, Mocko K, Martinez-Cabrera H, et al. Functional traits in parallel evolutionary radiations and trait-environment associations in the cape floristic region of South Africa. Am Nat. 2015;185(4):525-37.
28. Higgins SI, Scheiter S. Atmospheric CO_2 forces abrupt vegetation shifts locally, but not globally. Nature. 2012;488(7410):209-12.
29. Colgan MS, Martin RE, Baldeck CA, Asner GP. Tree foliar chemistry in an African savanna and its relation to life history strategies and environmental filters. PLoS One. 2015;10(5):e0124078-e.
30. Odgaard BV. Fossil pollen as a record of past biodiversity. J Biogeogr. 1999;26(1):7-17.
31. Eglinton TI, Eglinton G. Molecular proxies for paleoclimatology. Earth Planet Sc Lett. 2008;275:1-16.
32. Rommerskirchen F, Plader A, Eglinton G, Chikaraishi Y, Rullkötter J. Chemotaxonomic significance of distribution and stable carbon isotopic composition of long-chain alkanes and alkan-1-ols in C4 grass waxes. Org Geochem. 2006;37(10):1303-32. doi: 10.1016/j.orggeochem.2005.12.013.

33. Vogts A, Moossen H, Rommerskirchen F, Rullkötter J. Distribution patterns and stable carbon isotopic composition of alkanes and alkan-1-ols from plant waxes of African rain forest and savanna C3 species. Org Geochem. 2009;40(10):1037-54.
34. Bush RT, McInerney FA. Leaf wax n-alkane distributions in and across modern plants: implications for paleoecology and chemotaxonomy. Geochim Cosmochim Ac. 2013;117:161-79.
35. Magill CR, Ashley GM, Freeman KH. Ecosystem variability and early human habitats in eastern Africa. P Natl Acad Sci USA. 2013;110(4):1167-74.
36. Magill CR, Ashley GM, Domínguez-Rodrigo M, Freeman KH. Dietary options and behavior suggested by plant biomarker evidence in an early human habitat. P Natl Acad Sci USA. 2016;113(11):2874-9.
37. Hemingway JD, Schefuß E, Dinga BJ, Pryer H, Galy VV. Multiple plant-wax compounds record differential sources and ecosystem structure in large river catchments. Geochim Cosmochim Ac. 2016;184:20-40.
38. Bush RT, Wallace J, Currano ED, Jacobs BF, McInerney FA, Dunn RE, et al. Cell anatomy and leaf $\delta^{13}C$ as proxies for shading and canopy structure in a Miocene forest from Ethiopia. Palaeogeogr Palaeocl. 2017;485(Supplement C):593-604. doi: https://doi.org/10.1016/j.palaeo.2017.07.015.
39. Schefuß E, Kuhlmann H, Mollenhauer G, Prange M, Pätzold J. Forcing of wet phases in southeast Africa over the past 17,000 years. Nature. 2011;480(7378):509-12. doi: 10.1038/nature10685.
40. Wang YV, Larsen T, Leduc G, Andersen N, Blanz T, Schneider RR. What does leaf wax δD from a mixed C3/C4 vegetation region tell us? Geochim Cosmochim Ac. 2013;111:128-39. doi: 10.1016/j.gca.2012.10.016.
41. Westoby M, Falster DS, Moles AT, Vesk PA, Wright IJ. Plant ecological strategies: some leading dimensions of variation between species. Annu Rev Ecol Syst. 2002:125-59.
42. Magill CR, Denis EH, Freeman KH. Rapid sequential separation of sedimentary lipid biomarkers via selective accelerated solvent extraction. Org Geochem. 2015;88:29-34.
43. Sadler PM, Jerolmack DJ. Scaling laws for aggradation, denudation and progradation rates: the case for time-scale invariance at sediment sources and sinks. Geol Soc Spec Publ. 2014;404(1):SP404.7-SP.7. doi: 10.1144/SP404.7.
44. Brooks JR, Flanagan LB, Buchmann N, Ehleringer JR. Carbon isotope composition of boreal plants: functional grouping of life forms. Oecologia. 1997;110(3):301-11.
45. Chen S, Bai Y, Lin G, Han X. Variations in life-form composition and foliar carbon isotope discrimination among eight plant communities under different soil moisture conditions in the Xilin River Basin, Inner Mongolia, China. Ecol Res. 2005;20(2):167-76.
46. Lloyd J, Bird MI, Vellen L, Miranda AC, Veenendaal EM, Djagbletey G, et al. Contributions of woody and herbaceous vegetation to tropical savanna ecosystem productivity: a quasi-global estimate. Tree Physiol. 2008;28(3):451-68.
47. Krull ES, Skjemstad JO, Burrows WH, Bray SG, Wynn JG, Bol R, et al. Recent vegetation changes in central Queensland, Australia: evidence from $\delta^{13}C$ and 14C analyses of soil organic matter. Geoderma. 2005;126(3):241-59. doi: 10.1016/j.geoderma.2004.09.012.
48. Magill CR, Ashley GM, Freeman KH. Water, plants, and early human habitats in eastern Africa. P Natl Acad Sci USA. 2013;110(4):1175-80.
49. Kelly CK, Woodward FI. Ecological correlates of carbon isotope composition of leaves: a comparative analysis testing for the effects of temperature, CO2 and O2

partial pressures and taxonomic relatedness on $\delta^{13}C$. J Ecol. 1995;83(3):509-15. doi: 10.2307/2261603.
50. Breshears DD. The grassland-forest continuum: Trends in ecosystem properties for woody plant mosaics? Front Ecol Environ. 2006;4(Figure 2):96-104. doi: 10.1890/1540-9295(2006)004[0096:TGCTIE]2.0.CO;2.
51. Hüttich C, Herold M, Strohbach BJBJ, Dech S. Integrating in-situ, Landsat, and MODIS data for mapping in Southern African savannas: experiences of LCCS-based land-cover mapping in the Kalahari in Namibia. Environ Monit Assess. 2011;176(1-4):531-47.
52. De Vente J, Poesen J. Predicting soil erosion and sediment yield at the basin scale: scale issues and semi-quantitative models. Earth-Sci Rev. 2005;71(1):95-125.
53. Letters E, Gotelli NJ, Colwell RK, Letters E. Quantifying biodiversity : procedures and pitfalls in the measurement and comparison of species richness. Ecol Lett. 2001;4(May 1988):379-91.
54. Moya-Laraño J, Corcobado G. Plotting partial correlation and regression in ecological studies. Web Ecol. 2008;8(1):35-46. doi: 10.5194/we-8-35-2008.
55. Castañeda IS, Werne JP, Johnson TC, Filley TR. Late Quaternary vegetation history of southeast Africa: the molecular isotopic record from Lake Malawi. Palaeogeogr Palaeocl. 2009;275(1):100-12.
56. DeBusk GH. A 37,500-year pollen record from Lake Malawi and implications for the biogeography of afromontane forests. J Biogeogr. 1998;25:479-500.
57. Ivory SJ, Lézine A-M, Vincens A, Cohen AS. Effect of aridity and rainfall seasonality on vegetation in the southern tropics of East Africa during the Pleistocene/Holocene transition. Quaternary Res. 2012;77(1):77-86.
58. Ivory SJ, McGlue MM, Ellis GS, Lézine A-M, Cohen AS, Vincens A. Vegetation controls on weathering intensity during the last deglacial transition in southeast Africa. PLoS One. 2014;9(11):e112855-e.
59. Daru BH, Bank M, Maurin O, Yessoufou K, Schaefer H, Slingsby JA, et al. A novel phylogenetic regionalization of phytogeographical zones of southern Africa reveals their hidden evolutionary affinities. J Biogeogr. 2016;43(1):155-66.
60. Sugita S. Pollen representation of vegetation in Quaternary sediments: theory and method in patchy vegetation. J Ecol. 1994;82(4):881-97.
61. Mander L, Punyasena SW. On the taxonomic resolution of pollen and spore records of Earth's vegetation. Int J Plant Sci. 2014;175(8):931-45.
62. Ivory SJ, Russell J. Climate, herbivory, and fire controls on tropical African forest for the last 60ka. Quaternary Science Reviews. 2016;148(Supplement C):101-14. doi: https://doi.org/10.1016/j.quascirev.2016.07.015.
63. Margalef R, Gutierrez E. How to introduce connectance in the frame of an expression for diversity. Am Nat. 1983:601-7.
64. Timberlake J. Biodiversity of the Zambezi Basin. Occas Publ Biodivers. 2000;9(9):1-22.
65. Burrough SL, Willis KJ. Ecosystem resilience to late-Holocene climate change in the Upper Zambezi Valley. Holocene. 2015;25(11):1811-28. doi: 10.1177/0959683615591355.
66. Sexton JO, Song X-P, Feng M, Noojipady P, Anand A, Huang C, et al. Global, 30-m resolution continuous fields of tree cover: Landsat-based rescaling of MODIS vegetation continuous fields with lidar-based estimates of error. Int J Digit Earth. 2013;6(5):427-48. doi: 10.1080/17538947.2013.786146.
67. Ryan CM, Williams MM, Hill TC, Grace J, Woodhouse IH. Assessing the phenology of southern tropical Africa: a comparison of hemispherical photography, scatterometry,

and optical/NIR remote sensing. IEEE T Geosci Remote. 2014;52(1):519-28. doi: 10.1109/TGRS.2013.2242081.
68. Khon VC, Wang YV, Krebs-Kanzow U, Kaplan JO, Schneider RR, Schneider B. Climate and CO2 effects on the vegetation of southern tropical Africa over the last 37,000 years. Earth Planet Sc Lett. 2014;403(0):407-17. doi: http://dx.doi.org/10.1016/j.epsl.2014.06.043.
69. O'Connor TG, Haines LM, Snyman HA. Influence of precipitation and species composition on phytomass of a semi-arid African grassland. J Ecol. 2001;89(5):850-60. doi: 10.1046/j.0022-0477.2001.00605.x.
70. Schubert BA, Jahren AH. Global increase in plant carbon isotope fractionation following the Last Glacial Maximum caused by increase in atmospheric pCO2. Geology. 2015;43(5):435-8.
71. Nicotra ABAB, Atkin OKOK, Bonser SPSP, Davidson AM, Finnegan EJ, Mathesius U, et al. Plant phenotypic plasticity in a changing climate. Trends Plant Sci. 2010;15(12):684-92.
72. Dalerum F, Cameron EZ, Kunkel K, Somers MJ. Interactive effects of species richness and species traits on functional diversity and redundancy. Theor Ecol. 2012;5(1):129-39.
73. Anderson TM, Metzger KL, McNaughton SJ, Michael Anderson T, Metzger KL, McNaughton SJ. Multi-scale analysis of plant species richness in Serengeti grasslands. J Biogeogr. 2007;34(2):313-23. doi: 10.1111/j.1365-2699.2006.01598.x.
74. Franco AC, Rossatto DR, Silva LdCR, da Silva Ferreira C. Cerrado vegetation and global change: the role of functional types, resource availability and disturbance in regulating plant community responses to rising CO2 levels and climate warming. Theor Exp Plant Physiol. 2014;26(1):19-38.
75. Ainsworth EA, Long SP. What have we learned from 15 years of free-air CO2 enrichment (FACE)? A meta-analytic review of the responses of photosynthesis, canopy properties and plant production to rising CO_2. New Phytol. 2005;165(2):351-72.
76. Polley HW, Jin VL, Fay PA. CO_2-caused change in plant species composition rivals the shift in vegetation between mid-grass and tallgrass prairies. Glob Change Biol. 2012;18(2):700-10.
77. Callaway RM, Pennings SC, Richards CL. Phenotypic plasticity and interactions among plants. Ecology. 2003;84(5):1115-28.
78. Tsialtas JT, Handley LL, Kassioumi MT, Veresoglou DS, Gagianas AA. Interspecific variation in potential water-use efficiency and its relation to plant species abundance in a water-limited grassland. Funct Ecol. 2001;15(5):605-14.
79. Kahmen A, Perner J, Buchmann N. Diversity-dependent productivity in semi-natural grasslands following climate perturbations. Funct Ecol. 2005;19(4):594-601.
80. Silvertown J, Araya Y, Gowing D. Hydrological niches in terrestrial plant communities: a review. J Ecol. 2015;103(1):93-108. doi: 10.1111/1365-2745.12332.
81. Sala OE, Chapin FS, Armesto JJ, Berlow E, Bloomfield J, Dirzo R, et al. Global biodiversity scenarios for the year 2100. Science. 2000;287(5459):1770-4.
82. Cernusak LA, Ubierna N, Winter K, Holtum JAM, Marshall JD, Farquhar GD. Environmental and physiological determinants of carbon isotope discrimination in terrestrial plants. New Phytol. 2013;200(4):950-65.
83. Leakey ADB, Lau JA. Evolutionary context for understanding and manipulating plant responses to past, present and future atmospheric [CO2]. Philos T Roy Soc B. 2012;367(1588):613-29.

84. Busch FA, Sage TL, Cousins AB, Sage RF. C3 plants enhance rates of photosynthesis by reassimilating photorespired and respired CO2. Plant Cell Environ. 2013;36(1):200-12.
85. Kleidon A, Adams J, Pavlick R, Reu B. Simulated geographic variations of plant species richness, evenness and abundance using climatic constraints on plant functional diversity. Environ Res Lett. 2009;4(1):14007-.
86. Deryng D, Elliott J, Folberth C, Müller C, Pugh TAM, Boote KJ, et al. Regional disparities in the beneficial effects of rising CO2 concentrations on crop water productivity. Nature Clim Change. 2016;6:786-90.
87. Solomon S, Qin D, Manning M, Chen Z, Marquis M, Averyt KB, et al. Contribution of working group I to the fourth assessment report of the intergovernmental panel on climate change, 2007. Cambridge University Press, Cambridge; 2007.
88. Heubes J, Schmidt M, Stuch B, Márquez JRG, Wittig R, Zizka G, et al. The projected impact of climate and land use change on plant diversity: an example from West Africa. J Arid Environ. 2013;96:48-54.
89. Grace J, Jose JS, Meir P, Miranda HS, Montes RA. Productivity and carbon fluxes of tropical savannas. J Biogeogr. 2006;33(3):387-400. doi: 10.1111/j.1365-2699.2005.01448.x.
90. Gill RA, Polley HW, Johnson HB, Anderson LJ, Maherali H, Jackson RB. Nonlinear grassland responses to past and future atmospheric CO2. Nature. 2002;417(6886):279-82.
91. Jackson RB, Banner JL, Jobbágy EG, Pockman WT, Wall DH. Ecosystem carbon loss with woody plant invasion of grasslands. Nature. 2002;418(6898):623-6.
92. Anchukaitis KJ, Tierney JE. Identifying coherent spatiotemporal modes in time-uncertain proxy paleoclimate records. Clim Dynam. 2013;41(5-6):1291-306.
93. Monnin E, Indermühle A, Dällenbach A, Flückiger J, Stauffer B, Stocker TF, et al. Atmospheric CO2 concentrations over the last glacial termination. Science. 2001;291(5501):112-4.
94. Wang YV, Leduc G, Regenberg M, Andersen N, Larsen T, Blanz T, et al. Northern and southern hemisphere controls on seasonal sea surface temperatures in the Indian Ocean during the last deglaciation. Paleoceanography. 2013;28(4):619-32. doi: 10.1002/palo.20053.
95. White F. The vegetation of Africa: a descriptive memoir to accompany the UNESCO/AETFAT/UNSO vegetation map of Africa. Unesco, Paris; 1983.
96. Sankaran M, Hanan NP, Scholes RJ, Ratnam J, Augustine DJ, Cade BS, et al. Determinants of woody cover in African savannas. Nature. 2005;438(7069):846-9. doi: 10.1038/nature04070.
97. Cryer D. Epicuticular Waxes as Continental Paleovegetational Biomarkers 2012.
98. Bezabih M, Pellikaan WF, Tolera A, Hendriks WH. Evaluation of n-alkanes and their carbon isotope enrichments (δ13C) as diet composition markers. Animal. 2011;5(1):57-66. doi: 10.1017/S1751731110001515.
99. Bezabih M, Pellikaan WFF, Hendriks WHH. Using n-alkanes and their carbon isotope enrichments (δ13C) to estimate the botanical composition of pasture mixes from the Mid Rift Valley grasslands of Ethiopia. Livest Sci. 2011;142(1):298-304. doi: 10.1016/j.livsci.2011.08.009.
100. Zuur A, Ieno EN, Smith GM. Analysing Ecological Data: Springer Science & Business Media; 2007.
101. Ficken KJ, Li B, Swain DL, Eglinton G. An n-alkane proxy for the sedimentary input of submerged/floating freshwater aquatic macrophytes. Org Geochem. 2000;31(7):745-9.

102. Hansen MC, Potapov PV, Moore R, Hancher M, Turubanova SA, Tyukavina A, et al. High-resolution global maps of 21st-century forest cover change. Science. 2013;342(6160):850-3. doi: 10.1126/science.1244693.
103. Hill MJ, Román MO, Schaaf CB. Dynamics of vegetation indices in tropical and subtropical savannas defined by ecoregions and Moderate Resolution Imaging Spectroradiometer (MODIS) land cover. Geocarto Int. 2012;27(2):153-91.
104. Kögel-Knabner I. The macromolecular organic composition of plant and microbial residues as inputs to soil organic matter. Soil Biol Biochem. 2002;34(2):139-62.
105. Guan K, Wood EF, Caylor KK. Multi-sensor derivation of regional vegetation fractional cover in Africa. Remote Sens Environ. 2012;124:653-65.
106. Teisserenc R, Lucotte M, Houel S, Carreau J. Integrated transfers of terrigenous organic matter to lakes at their watershed level: a combined biomarker and GIS analysis. Geochim Cosmochim Ac. 2010;74(22):6375-86.
107. Beilfuss R, Dos Santos D. Patterns of hydrological change in the Zambezi Delta, Mozambique. 2001.
108. Kristen I, Wilkes H, Vieth A, Zink KG, Plessen B, Thorpe J, et al. Biomarker and stable carbon isotope analyses of sedimentary organic matter from Lake Tswaing: evidence for deglacial wetness and early Holocene drought from South Africa. J Paleolimnol. 2010;44(1):143-60.
109. Martins O, Probst J. Biogeochemistry of major African rivers: carbon and mineral transport. In: Degens ET, Kempe S, Richey JE, editors.: John Wiley & Sons; 1991. p. 127-55.
110. Vogts A, Schefuß E, Badewien T, Rullkötter J. n-Alkane parameters from a deep sea sediment transect off southwest Africa reflect continental vegetation and climate conditions. Org Geochem. 2012;47:109-19.
111. Dupont LM, Wyputta U. Reconstructing pathways of aeolian pollen transport to the marine sediments along the coastline of SW Africa. Quaternary Sci Rev. 2003;22(2-4):157-74. doi: 10.1016/S0277-3791(02)00032-X.
112. Bouimetarhan I, Dupont LM, Kuhlmann H, Pätzold J, Prange M, Schefuβ E, et al. Northern Hemisphere control of deglacial vegetation changes in the Rufiji uplands (Tanzania). Clim Past. 2015;11(5):751-64. doi: 10.5194/cp-11-751-2015.
113. Barkan J, Kutiel H, Alpert P, Kishcha P. Synoptics of dust intrusion days from the African continent into the Atlantic Ocean. J Geophys Res-Atmos. 2004;109(D08201).
114. Schulz H, Lückge A, Emeis K-C, Mackensen A. Variability of Holocene to Late Pleistocene Zambezi riverine sedimentation at the upper continental slope off Mozambique, 15°–21°S. Mar Geol. 2011;286(1):21-34. doi: 10.1016/j.margeo.2011.05.003.
115. Schefuß E, Ratmeyer V, Stuut J-BW, Jansen JHF, Damsté JSS. Carbon isotope analyses of n-alkanes in dust from the lower atmosphere over the central eastern Atlantic. Geochim Cosmochim Ac. 2003;67(10):1757-67.
116. Draxler R, Rolph G. HYSPLIT (HYbrid Single-Particle Lagrangian Integrated Trajectory). 2003.
117. Lepple FK, Brine CJ. Organic constituents in eolian dust and surface sediments srom northwest Africa. J Geophys Res. 1976;81(6):1141-7.
118. Simoneit BR. Organic matter in eolian dusts over the Atlantic Ocean. Mar Chem. 1977;5(4-6):443-64.
119. Herrmann N, Boom A, Carr AS, Chase BM, Granger R, Hahn A, et al. Sources, transport and deposition of terrestrial organic material: A case study from southwestern Africa. Quaternary Sci Rev. 2016;149:215-29.

120. Eglinton TI, Eglinton G, Dupont L, Sholkovitz ER, Montluçon D, Reddy CM. Composition, age, and provenance of organic matter in NW African dust over the Atlantic Ocean. Geochem Geophys Geosy. 2002;3(8):1-27.
121. Rommerskirchen F, Eglinton G, Dupont L, Güntner U, Wenzel C, Rullkötter J. A north to south transect of Holocene southeast Atlantic continental margin sediments: Relationship between aerosol transport and compound-specific δ13C land plant biomarker and pollen records. Geochem Geophys Geosy. 2003;4(1101):20-. doi: 10.1029/2003GC000541.
122. Garcin Y, Schefuß E, Schwab VF, Garreta V, Gleixner G, Vincens A, et al. Reconstructing C3 and C4 vegetation cover using n-alkane carbon isotope ratios in recent lake sediments from Cameroon, Western Central Africa. Geochim Cosmochim Ac. 2014;142:482-500. doi: 10.1016/j.gca.2014.07.004.
123. West JB, Bowen GJ, Dawson TE, Tu KP. Isoscapes: Understanding Movement, Pattern, and Process on Earth Through Isotope Mapping. New York: Springer; 2010. 495- p.
124. Sachse D, Billault I, Bowen GJ, Chikaraishi Y, Dawson TE, Feakins SJ, et al. Molecular paleohydrology: Interpreting the hydrogen-isotopic composition of lipid biomarkers from photosynthesizing organisms. Annu Rev Earth Pl Sc. 2012;40:221-49.
125. Crawley MJ, Harral JE. Scale dependence in plant biodiversity. Science. 2001;291(5505):864-8.
126. Adler PB, White EP, Lauenroth WK, Kaufman DM, Rassweiler A, Rusak JA. Evidence for a general species-time-area relationship. Ecology. 2005;86(8):2032-9.
127. Safford HD, Rejmánek M, Hadač E. Species pools and the "hump-back" model of plant species diversity: an empirical analysis at a relevant spatial scale. Oikos. 2001;95(2):282-90.
128. Kindt R, Lillesø JPB, Breugel P, Bingham M, Demissew S, Dudley C, et al. Correspondence in forest species composition between the Vegetation Map of Africa and higher resolution maps for seven African countries. Appl Veg Sci. 2014;17(1):162-71.
129. Adler PB, Lauenroth WK. The power of time: spatiotemporal scaling of species diversity. Ecol Lett. 2003;6(8):749-56.
130. Levin S. The problem of pattern and scale in ecology. Ecology. 1992;73(6):1943-67.
131. Blarquez O, Finsinger W, Carcaillet C. Assessing paleo-biodiversity using low proxy influx. PLoS One. 2013;8(6):e65852-e.
132. Küper W, Sommer JH, Lovett JC, Barthlott W. Deficiency in African plant distribution data–missing pieces of the puzzle. Bot J Linn Soc. 2006;150(3):355-68.
133. Gillison AN, Asner GP, Fernandes EC, Mafalacusser J, Banze A, Izidine S, et al. Biodiversity and agriculture in dynamic landscapes: Integrating ground and remotely-sensed baseline surveys. Journal of environmental management. 2016;177:9-19.
134. Diefendorf AF, Mueller KE, Wing SL, Koch PL, Freeman KH. Global patterns in leaf 13C discrimination and implications for studies of past and future climate. P Natl Acad Sci USA. 2010;107(13):5738-43.
135. O'Brien EM, Field R, Whittaker RJ. Climatic gradients in woody plant (tree and shrub) diversity: water-energy dynamics, residual variation, and topography. Oikos. 2000;89(3):588-600.
136. Stein A, Gerstner K, Kreft H. Environmental heterogeneity as a universal driver of species richness across taxa, biomes and spatial scales. Ecol Lett. 2014;17(7):n/a-n/a. doi: 10.1111/ele.12277.

137. Fant C, Gebretsadik Y, McCluskey A, Strzepek K. An uncertainty approach to assessment of climate change impacts on the Zambezi River Basin. Climatic Change. 2015;130(1):35-48. doi: 10.1007/s10584-014-1314-x.
138. Jung G, Prange M, Schulz M. Influence of topography on tropical African vegetation coverage. Clim Dynam. 2015;46(7):2535-49.
139. Brovkin V, Hofmann M, Bendtsen J, Ganopolski A. Ocean biology could control atmospheric δ13C during glacial-interglacial cycle. Geochem Geophys Geosy. 2002;3(5):1-15.
140. Kohn MJ. Carbon isotope discrimination in C3 land plants is independent of natural variations in pCO2. Geochem Perspect-Lett. 2016;2:35-43.
141. Parrenin F, Masson-Delmotte V, Köhler P, Raynaud D, Paillard D, Schwander J, et al. Synchronous change of atmospheric CO2 and Antarctic temperature during the last deglacial warming. Science. 2013;339(6123):1060-3. doi: 10.1126/science.1226368.
142. van der Lubbe JJL, Frank M, Tjallingii R, Schneider RR. Neodymium isotope constraints on provenance, dispersal, and climate-driven supply of Zambezi sediments along the Mozambique Margin during the past ~45,000 years. Geochem Geophys Geosy. 2015;17:181-98.
143. van der Lubbe JJL, Tjallingii R, Prins MA, Brummer G-JA, Jung SJAA, Kroon D, et al. Sedimentation patterns off the Zambezi River over the last 20,000years. Mar Geol. 2014;355:189-201. doi: 10.1016/j.margeo.2014.05.012.
144. Gillison A, Asner G, Richey J, Fernandes E. Lower Zambezi River Basin: Baseline Data on Land-use, Biodiversity and Hydrology. 2006.
145. Werger MJA, Coetzee BJ. 10 The Sudano-Zambezian Region ·. 1978;(December 1976).
146. O'Connor TG, Haines LM, Snyman HA. Influence of precipitation and species composition on phytomass of a semi-arid African grassland. J Ecol. 2001;89(5):850-60.
147. Wiles E, Green A, Watkeys M, Jokat W. Zambezi continental margin: compartmentalized sediment transfer routes to the abyssal Mozambique Channel. Marine Geophysical Research. 2017;38(3):227-40.
148. Just J, Schefuß E, Kuhlmann H, Stuut J-BW, Pätzold J. Climate induced sub-basin source-area shifts of Zambezi River sediments over the past 17ka. Palaeogeogr Palaeocl. 2014;410:190-9.
149. Romans BW, Castelltort S, Covault JA, Fildani A, Walsh JPJP. Environmental signal propagation in sedimentary systems across timescales. Earth-Sci Rev. 2015;153:7-29. doi: 10.1016/j.earscirev.2015.07.012.
150. Gasse F, Chalié F, Vincens A, Williams MAJ, Williamson D. Climatic patterns in equatorial and southern Africa from 30,000 to 10,000 years ago reconstructed from terrestrial and near-shore proxy data. Quaternary Sci Rev. 2008;27(25):2316-40.
151. Chevalier M, Chase BM. Southeast African records reveal a coherent shift from high-to low-latitude forcing mechanisms along the east African margin across last glacial–interglacial transition. Quaternary Sci Rev. 2015;125:117-30.
152. Campo EV, Duplessy JC, Prell WL. Comparison of terrestrial and marine temperature estimates for the past 135 kyr off southeast Africa: a test for GCM simulations of palaeoclimate. Nature. 1990.
153. Collatz GJ, Berry JA, Clark JS. Effects of climate and atmospheric CO2 partial pressure on the global distribution of C4 grasses: present, past, and future. Oecologia. 1998;114(4):441-54.
154. Vörösmarty CJ, Moore Iii B, Moore B. Modeling basin-scale hydrology in support of physical climate and global biogeochemical studies: An example using the Zambezi River. Surv Geophys. 1991;12(1-3):271-311. doi: 10.1007/BF01903422.

155. Davies BR, Beilfuss RD, Thoms MC. Cahora Bassa retrospective , 1974 – 1997 : effects of flow regulation on the Lower Zambezi River. Limnology in developing world. 2000;(December):1-9.
156. Nones M, Ronco P, Di Silvio G. Modelling the impact of large impoundments on the Lower Zambezi River. Int J River Basin Manag. 2013;11(2):221-36.
157. Ronco P, Fasolato G, Nones M, Di Silvio G. Morphological effects of damming on lower Zambezi River. Geomorphology. 2010;115(1):43-55.
158. Lambert T, Teodoru CR, Nyoni FC, Bouillon S, Darchambeau F, Massicotte P, et al. Along-stream transport and transformation of dissolved organic matter in a large tropical river. Biogeosciences. 2016;13:2727-41.
159. Mason NWHNWH, Mouillot D, Lee WGWG, Wilson JBB. Functional richness, functional evenness and functional divergence: the primary components of functional diversity. Oikos. 2005;1(1):112-8. doi: 10.1111/j.0030-1299.2005.13886.x.
